# A New Expression System Based on Psychrotolerant *Debaryomyces macquariensis* Yeast and Its Application to the Production of Cold-Active β-d-Galactosidase from *Paracoccus* sp. 32d

**DOI:** 10.3390/ijms231911691

**Published:** 2022-10-02

**Authors:** Marta Wanarska, Ewelina Krajewska-Przybyszewska, Monika Wicka-Grochocka, Hubert Cieśliński, Anna Pawlak-Szukalska, Aneta M. Białkowska, Marianna Turkiewicz, Tomasz Florczak, Ewa Gromek, Joanna Krysiak, Natalia Filipowicz

**Affiliations:** 1 Department of Molecular Biotechnology and Microbiology, Faculty of Chemistry, Gdansk University of Technology, Narutowicza 11/12, 80-233 Gdansk, Poland; 2 Institute of Molecular and Industrial Biotechnology, Faculty of Biotechnology and Food Sciences, Lodz University of Technology, Stefanowskiego 2/22, 90-573 Lodz, Poland

**Keywords:** *Debaryomyces macquariensis* strain D50, psychrotolerant yeast, host/vector expression system, constitutive *GAP* promoter, constitutive *TEF1* promoter, *CYC1* transcriptional terminator

## Abstract

Yeasts provide attractive host/vector systems for heterologous gene expression. The currently used yeast-based expression platforms include mesophilic and thermotolerant species. A eukaryotic expression system working at low temperatures could be particularly useful for the production of thermolabile proteins and proteins that tend to form insoluble aggregates. For this purpose, an expression system based on an Antarctic psychrotolerant yeast *Debaryomyces* *macquariensis* strain D50 that is capable of growing at temperatures ranging from 0 to 30 °C has been developed. The optimal physical culture conditions for *D. macquariensis* D50 in a fermenter are as follows: temperature 20 °C, pH 5.5, aeration rate of 1.5 vvm, and a stirring speed of 300 rpm. Four integrative plasmid vectors equipped with an expression cassette containing the constitutive *GAP* promoter and *CYC1* transcriptional terminator from *D. macquariensis* D50 were constructed and used to clone and express a gene-encoding cold-active β-d-galactosidase of *Paracoccus* sp. 32d. The yield was 1150 U/L of recombinant yeast culture. Recombinant *D. macquariensis* D50 strains were mitotically stable under both selective and non-selective conditions. The *D. macquariensis* D50 host/vector system has been successfully utilized for the synthesis of heterologous thermolabile protein, and it can be an alternative to other microbial expression systems.

## 1. Introduction

Currently, enzymes and other proteins are used in a wide range of applications, including biopharmaceuticals (monoclonal antibodies, clotting factors, hormones, enzymes, and vaccines), diagnostic proteins, detergent constituents, and enzymes used as catalysts in many different industrial processes. The global biopharmaceuticals market reached USD 186 billion in 2017 [1], whereas the global industrial enzyme market was USD 6 billion in 2017 [2]. The largest segment of industrial enzyme applications is the food industry. Enzymes are widely used for the production of dairy, bakery, and meat products and beverages, such as fruit juice, beer, and wine. Moreover, they are used in a series of technical applications, such as textile production, pulp and paper manufacturing, and biofuels production. According to the European Association of Manufacturers and Formulators of Enzyme Products (https://amfep.org, accessed on 12 May 2015), enzymes are used to make and improve nearly 400 everyday consumer and commercial products.

By using recombinant DNA technology, many proteins, including enzymes, can be produced in different prokaryotic and eukaryotic cell factories, such as bacteria [1,3,4], yeasts [1,3,5,6,7], filamentous fungi [1,2,8], microalgae [1,9,10,11], insect cells [1,3,12], mammalian cells [1,3,13], transgenic plants [1,3,14], and transgenic animals [1,3,15]. The list of commercial enzymes published by AMFEP (updated May 2015) includes 243 enzymes for food, feed, and technical applications, 114 of which are recombinant [2].

The bacterial systems of the production of recombinant proteins offer fast growth, easy propagation, short production time, high scale-up capacity, medium product yield, and low overall costs. *Escherichia coli* is a preferred expression host due to its well-known biochemistry and genetics, as well as simple and straightforward genetic manipulations. Some disadvantages of this prokaryotic expression system include a lack of posttranslational modifications of recombinant proteins (i.e., glycosylation, formation of disulfide bonds, phosphorylation, and proteolytic processing), formation of insoluble inclusion bodies, poor secretion, codon bias, and endotoxin issues. Thus, the *E. coli* expression system is constantly being improved to overcome these problems [1,3,16]. Recombinant proteins have also been produced in other bacterial cell factories, e.g., *Bacillus* spp., *Pseudomonas* spp., and *Streptomyces* spp., allowing the efficient secretion of recombinant proteins or lactic acid bacteria with the Generally Recognized as Safe status by FDA, such as *Lactobacillus* spp. and *Lactococcus lactis* [1,4,17].

Eukaryotic cells possess an endoplasmic reticulum and Golgi apparatus, which play a substantial role in posttranslational modifications and transport of proteins, and thus are widely used in the production of recombinant protein-based biopharmaceuticals. Among all first-time approved biopharmaceuticals from 2015 to July 2018, over half (53%) were monoclonal antibodies (mAbs), and, consequently, mammalian cells dominated the other eukaryotic expression systems [18]. Mammalian cells, including the most commonly used Chinese hamster ovary cells (CHO), have the ability to produce large and complex proteins with appropriate posttranslational modifications, especially glycosylation, with medium-to-high yields. However, the mammalian cell factories have some limitations, namely, the introduction of heterologous gene and selection of the clone being time-consuming, the risk of infection by viruses being high, the scale-up capacity being low, the propagation being difficult, the production time being long, and the ultimate overall costs being high [1,3].

For these reasons, yeasts are valuable eukaryotic hosts for the production of recombinant proteins for medical and industrial purposes. Yeasts combine advantages of prokaryotic and higher eukaryotic expression hosts, such as simple genetic manipulations, fast growth, low nutritional demands, scalable cultivation, good protein folding and secretion, and the ability to provide posttranslational modifications. In addition, yeasts provide high biomass concentrations, high product yields, and safe pathogen-free production, with medium overall costs [1,3]. The yeast *Saccharomyces cerevisiae* was first implemented as a host for recombinant protein production in 1981 [19]. It is still widely used in the production of biopharmaceuticals due to its GRAS status [18], although it has some disadvantages, e.g., fermentative metabolism, plasmid instability, quite low protein yield, and hypermannosylation of recombinant glycoproteins, which makes them highly immunogenic for humans [6,7]. These limitations have resulted in the development of alternative yeast expression systems, including *Komagataella phaffii* (*Pichia pastoris*) [6,7,20] *Ogataea polymorpha* (*Hansenula polymorpha*) [7,21], *Kluyveromyces lactis* [22], *Yarrowia lipolytica* [6,7,23], and *Blastobotrys adeninivorans* (*Arxula adeninivorans*) [24,25]. Methylotrophic yeast *K. phaffii* is currently considered to be one of the most effective and versatile platforms for the production of recombinant proteins, including biopharmaceuticals and industrial enzymes [6,26]. Moreover, it has recently started to gain attention as a model organism in fundamental research [27]. Most of the yeast expression hosts mentioned are mesophiles, with the exception of *O. polymorpha* and *B. adeninivorans*, which are thermotolerants [21,25].

There is not one universal cell factory capable of producing all possible recombinant proteins in a fast, efficient, cheap, and accurate manner, with respect to folding and biological activity. Therefore, the choice of a suitable expression system for the biosynthesis of a recombinant protein, especially the difficult-to-express protein, is very important. Incorrect folding can lead to protein degradation by host proteases or the formation of insoluble aggregates (inclusion bodies). Moreover, some proteins can be unstable (thermolabile) or toxic to the host cell. It was found that some of these problems can be solved by decreasing the temperature of the cultivation of the mesophilic expression host, e.g., *E. coli*. Unfortunately, the major disadvantage of culturing *E. coli* at low temperatures is the significant reduction in biomass productivity. Thus, the *E. coli* ArcticExpress and ArcticExpress (DE3) strains (Stratagene, an Agilent Technologies division, USA), with constitutive expression of *cpn10* and *cpn60* genes for cold-adapted chaperonins from *Oleispira antarctica*, have been developed to enhance growth and folding at low temperatures, and increase soluble protein yields. However, the co-purification of Cpn10 and Cpn60 with the protein of interest is a common problem [16]. Therefore, the most promising strategy for the efficient production of difficult proteins at low temperatures appears to be the use of naturally cold-adapted expression hosts. To date, cold-adapted bacteria, such as *Pseudoalteromonas haloplanktis* TAC125 [28,29,30,31], *Pseudoalteromonas* sp. strain SM20429 [32], *Shewanella livingstonensis* Ac10 [33,34], *Rhodococcus erythropolis* strain JCM3201 [35], and *Photobacterium profundum* strain SS9 [36], have been employed for recombinant protein production at low temperatures. The microbial cell factories developed on the basis of psychrotolerant hosts are especially useful for the production of cold-active and thermolabile enzymes for research and industrial applications.

In this study, we developed a new expression system using a psychrotolerant Antarctic yeast, *Debaryomyces macquariensis* strain D50, as the host. We report the isolation and characterization of this strain; the construction of expression vectors; and the production of a foreign protein, a cold-active β-d-galactosidase from psychrotolerant bacterium *Paracoccus* sp. 32d [37], using this newly developed eukaryotic cell factory.

## 2. Results

### 2.1. Identification and General Characterization of D50 Strain

**Figure 1:**
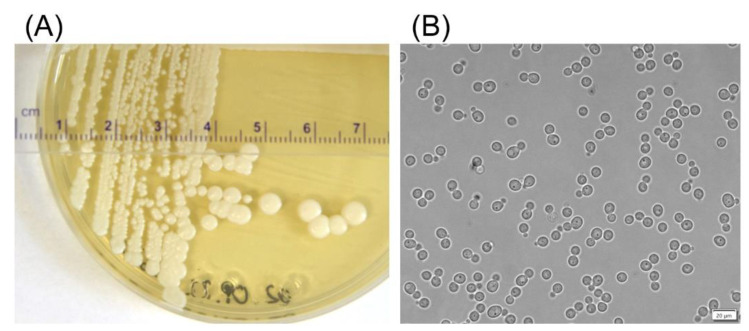
The yeast D50 strain grown at 20 °C on YPD agar plate for 7 days (**A**) and in liquid YPD at 150 rpm for 2 days (**B**).

The yeast strain D50 was isolated from an Antarctic soil sample. On the YPD-agar medium, it produced round opaque and convex cream-colored colonies with a smooth surface (Figure 1A). Cells of D50 yeast are round and budding (Figure 1B), they do not form pseudomycelium. In API 20 C AUX tests, the strain utilized a variety of common carbon sources, including glucose, galactose, xylose, l-arabinose, maltose, sucrose, trehalose, cellobiose, raffinose, melezitose, sorbitol, xylitol, adonitol, glycerol, calcium 2-keto-gluconate, methyl α-d-glucopyranoside, and N-acetyl-glucosamine; it did not utilize inositol and lactose. The yeast has a weak ability to ferment glucose and does not ferment galactose, sucrose, trehalose, and raffinose (API Candida test). The D50 strain was able to assimilate ammonium sulfate, ethylamine hydrochloride, and cadaverine dihydrochloride. The utilization of l-lysine was weak. The other nitrogen sources, namely potassium nitrate, sodium nitrite, creatine, creatinine monohydrate, d-glucosamine, imidazole, and d-tryptophan, were not assimilated. In the API Zym test, the D50 strain showed only weak activity of C4 esterase, C8 esterase/lipase, and leucine arylamidase. No alkaline and acid phosphatase, lipase (C14), α- and β-galactosidase, β-glucuronidase, α- and β-glucosidase, N-acetyl-β-glucosaminidase, α-mannosidase, or α-fucosidase activities were found. In the plate test, the strain D50 exhibited weak proteolytic activity (5 mm halo after 4 days of growth on YPD-agar medium with defatted milk at 20 °C). Extracellular proteolytic activity after 2- and 3-days cultivation of the strain in liquid YPD medium with casein was also small (3.8–6.3 and 5.7–7.5 nmol/mL × min at pH 7.0 and 4.0, respectively). The strain was found to grow at NaCl concentrations ranging from 1 to 10%. During a 48–72 h cultivation in liquid YPD strain, D50 secrete to the medium relatively small amounts of protein, about 0.025–0.030 mg/mL. For comparison, the proteolytic activity and concentration of extracellular proteins of yeast strains isolated from soil samples collected from various sampling sites in Antarctica are shown in Table S1. The yeast D50 strain was able to grow on YPD-agar medium supplemented with the antibiotic G418, even at very high concentration of 1000 μg/mL, and it was unable to grow on the medium supplemented with the antibiotic Zeocin, even at a low concentration of 25 μg/mL. Given the optimum and maximum growth temperatures of 20 and 30 °C, respectively, D50 was classified as a psychrotolerant microorganism. Cell growth was also observed at 0 °C, which was the lowest temperature tested.

**Figure 2:**
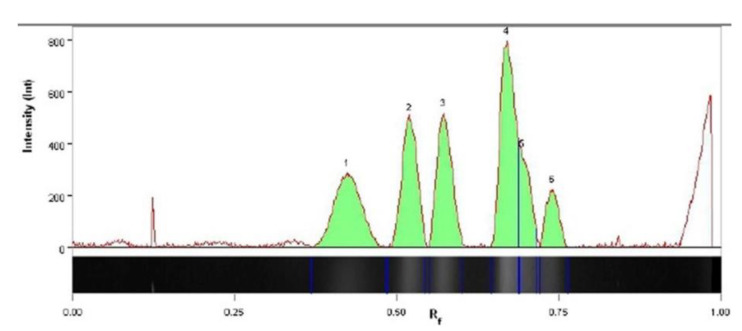
Pulsed field gel electrophoresis of *Debaryomyces macquariensis* strain D50 genomic DNA. The *Hansenula wingei* chromosomal DNA (Bio-Rad, Hercules, CA, USA) was used as size marker.

Based on the alignment of the D1/D2 domains of the large subunit (LSU) rRNA gene sequence and the ITS1-ITS2 regions of the rRNA gene (GenBank, accession no. ON759337 and ON759420, respectively) with the sequences available in GenBank database at NCBI, the D50 strain was classified as *Debaryomyces macquariensis* (99.81% sequence similarity in the D1/D2 regions (one substitution) with these of the *Debaryomyces macquariensis* type strain CBS 5572^T^ (FR799729 or NG_055693) and *D. macquariensis* IBT-D11 (KT313765), and 100% identity in the ITS1-ITS2 sequences (AM992909 and KT323950, respectively)). The *D. macquariensis* strain D50 has been deposited as KKP 2066p in the Culture Collection of Industrial Microorganisms maintained by the Prof. Wacław Dąbrowski Institute of Agriculture and Food Biotechnology (Warsaw, Poland). Furthermore, flow cytometric and pulsed-field gel electrophoretic analysis of the strain revealed an estimated genome size of 12.14 ± 0.20 and 11.426 Mb, respectively, with haploid (FCM/PFGE = 1.063) six chromosomes (Figure 2). The sizes of the chromosomes were as follows: 2.739, 2.318, 2.033, 1.634, 1.484, and 1.218 Mb.

### 2.2. Optimization of Culture Conditions for the D. macquariensis Strain D50 to Maximize the Growth

The medium with a composition of 5% glucose and 2% yeast extract was the most optimal in terms of the ratio of *D. macquariensis* D50 biomass growth (OD_660_) to time equal to 12.5. Under these conditions, the extracellular proteolytic activity was minimal (about 4 nmol/mL × min), and the protein concentration in the culture liquid did not exceed 0.02 mg/mL. Electrophoregrams made for the culture liquid did not show the presence of extracellular proteins. In the next step, Taguchi’s mathematical method was used to optimize the physical parameters of the process to maximize yeast strain D50 growth. The response was the growth of *D. macquariensis* determined by measuring the turbidity of the post-culture fluid at 660 nm (Table 1). Three levels of four input factors (Table 8 in the Materials and Methods section) were tested in an L_9_ array (Table 9 in the Materials and Methods section), and the trials were performed in four replicates. The experimental data were analyzed using MiniTab 18 software. The mean S/N coefficient was calculated for each run using the formula on the higher-the-better principles (Table 1). The values of the S/N ratio of individual levels for each studied factor were calculated and shown in Table 2. Those data were used for the optimum process conditions determination. The factor-level combination at which the growth of the *D. macquariensis* strain D50 is optimal is A1, B1, C3, and D3, i.e., temperature 20 °C, pH 5.5, aeration 1.5 vvm, and stirring 300 rpm.

Next, the analysis of variance (ANOVA) was used to determine the significant parameters affecting biomass production and its results are summarized in Table 3.

**Figure 3:**
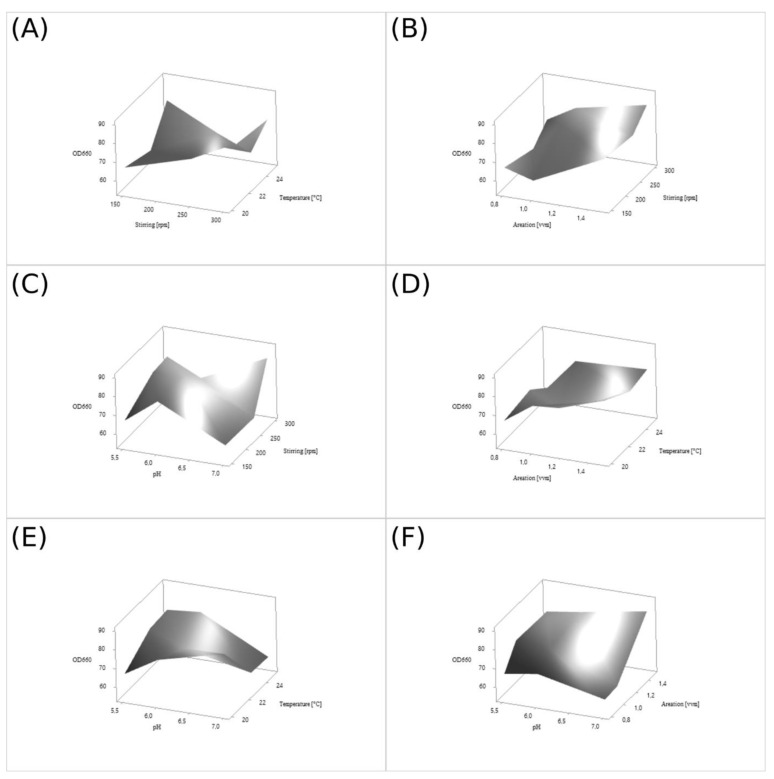
**The** 3D response plots and corresponding contour plots of yeast biomass production. The correlation between the physical parameters of culture: stirring and temperature (**A**); aeration and stirring (**B**); pH and stirring (**C**); aeration and temperature (**D**); pH and temperature (**E**); pH and aeration (**F**); and the biomass growth (OD_660_). The maximum values are highlighted.

The value of the model F-statistic of 8.42 implies that the model is significant. The multiple correlation coefficient R^2^ was over 0.71, which explains the 71% variation of the response. The calculated *p*-values suggest that the temperature, stirring, and aeration have a significant impact on biomass production at a 95% confidence level, while the process was not affected by the pH of the culture medium. The percentage influence of the factors on growth of strain D50 is found to be in the following order: aeration (42.5%) > stirring (13.6%) > temperature (8.9%) > pH (6.4%). Less than 29% of the contribution was error, which can be result of different variables uncontrolled in this study. The curves presented in Figure 3A–F show the impacts of each pair of factors. The 3D response surface plots were drawn to illustrate the combined effects of stirring and temperature (Figure 3A), aeration and stirring (Figure 3B), pH and stirring (Figure 3C), aeration and temperature (Figure 3D), pH and temperature (Figure 3E), and pH and aeration (Figure 3F). The theoretical value of biomass production (OD_660_ 91.26) in optimized conditions was determined by transforming formula (1) using the theoretical optimum process S/N coefficient calculated from formula (2). Those calculations were verified by culturing *D. macquariensis* strain D50 in the conditions chosen on the basis of Table 2 in four replicates. The average experimental biomass production was OD_660_ 88.20, which is consistent with the theoretical value obtained by the Taguchi method (1-sample T-student test, *p* = 0.36). The presented process of physicochemical culture conditions optimization led to an approximately 25% increase in biomass production, as compared to the standard conditions (OD_660_ 70).

### 2.3. Sequencing and Analysis of the D. macquariensis Strain D50 Genome

The genome of *D. macquariensis* strain D50 was sequenced using MiSeq System (Illumina, San Diego, CA, USA) at Genomed S.A. (Warsaw, Poland). The resulting assembly of yeast genome sequence has a length of 11,368,350 bp in 128 scaffolds with an *N*_50_ scaffold length of 746,630 bp. The draft genome has 34.79% G + C content. The draft genome encodes 202 putative tRNAs (without isotypes) and 4 pseudogenes. Moreover, gene prediction and annotation resulted in 5758 CDS encoding putative proteins, including 5204 proteins with predicted function. The maximum and minimum lengths of predicted proteins sequences are 4991 and 27 amino acids, respectively. For comparison, the G + C content of salt-tolerant *Debaryomyces hansenii* var. *hansenii* strain MTCC 234 isolated from New Zealand soil is 35.42%. The bioinformatics analysis of the draft genome sequence of *D. hansenii* var. *hansenii* strain MTCC 234 revealed 5313 predicted proteins, including 5069 proteins mapped to the UniProt database. Moreover, the maximum and minimum lengths of predicted proteins sequences are 4972 and 22 amino acids, respectively [38].

For constructing the series of yeast expression vectors, we decided to use the putative constitutive promoters of the *D. macquariensis* D50 *TEF1* (translation elongation factor 1α) and *GAP* (glyceraldehyde-3-phosphate dehydrogenase) genes. To this end, we searched the automated annotation results of the draft genome sequence of *D. macquariensis* D50 and identified the putative *TEF1* and *GAP* gene sequences (located in a sequence of scaffold 33). Next, the manual annotation confirmed these search results, however, it also revealed the presence of the alternative *TEF1* gene sequence (located in a sequence of scaffold 19), which we called *IEF2*. After the localization of *TEF1*, *IEF2*, and *GAP* genes, the noncoding DNA sequences located upstream of them were analyzed for the presence of TATA-box or TATA-like element sequences using the YAPP eukaryotic core promoter predictor. In all analyzed sequences, the putative TATA-box or TATA-like sequences with a score above the default score equal to 0.8 (cutoff score) were predicted. Next, the prediction results analysis based on the criteria presented in Materials and Methods allowed us to estimate the length of the DNA sequence, which contains the putative core promoter region for each analyzed *D. macquariensis* D50 gene, i.e., 576 bp for *TEF1* (Figure S1), 659 bp for *IEF2* gene (Figure S2), and 500 bp for *GAP* gene (Figure S3). Moreover, the putative transcription termination region (266 bp) of the *D. macquariensis* D50 *CYC1* (cytochrome C isoform 1) gene was also analyzed (Figure S4). The *CYC1* gene was localized during the automatic annotation of the *D. macquariensis* D50 draft genome sequence and confirmed with the manual one. It was located in a sequence of scaffold 39.

In addition, in this study, we also decided to exploit the usefulness of genetic elements derived from other psychrotolerant yeast *Candida santamariae* strain G12. For this purpose, we tested the putative promoter of the *C. santamariae* G12 *TEF1* gene (339 bp; Figure S5) and the putative transcriptional terminator of the *CYC1* gene (564 bp; Figure S6). Both genetic elements were predicted in our counterpart study on the *C. santamariae* strain G12 isolated from Baltic Sea water.

### 2.4. Construction of Expression Vectors

**Figure 4:**
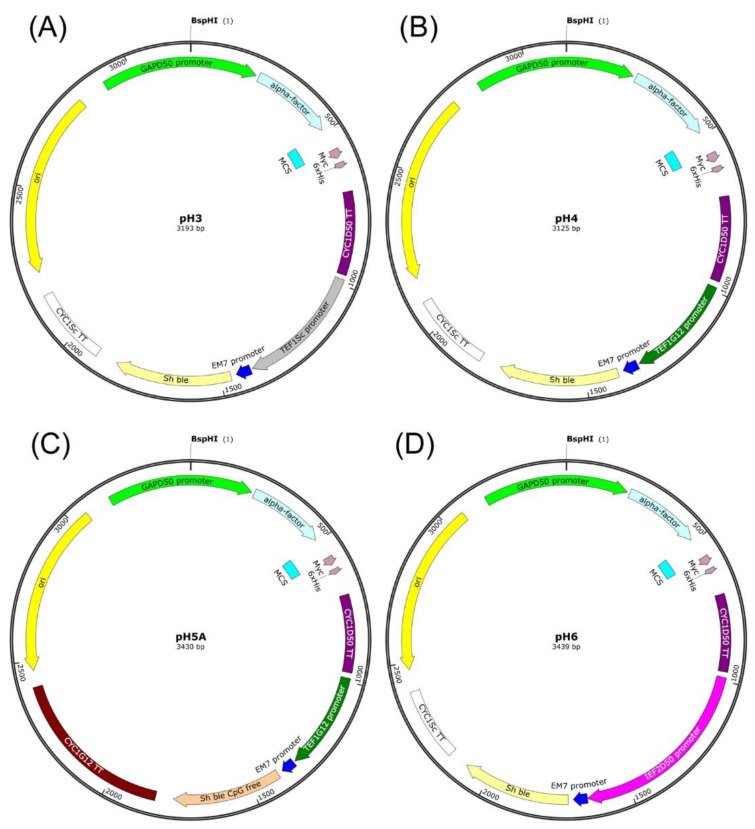
Maps of shuttle expression/integration vectors pH3, pH4, pH5A, and pH6 used in this study. All vectors contain the following elements: the pUC origin of replication (ori) for maintenance of plasmids in *E. coli*, the expression cassette consisting of *D. macquariensis* D50-derived *GAP* promoter (GAPD50 promoter), the sequence encoding *S. cerevisiae*-derived α-mating factor signal peptide (alpha-factor), multiple cloning site (MCS), C-terminal *myc* epitope (Myc), C-terminal polyhistidine tag (6xHis), and the *D. macquariensis* D50-derived *CYC1* transcription termination region (CYC1D50 TT). The pH3 vector (**A**) also contains the selection marker expression cassette composed of *S. cerevisiae*-derived *TEF1* promoter (TEF1Sc promoter), synthetic prokaryotic promoter that drives expression of the *Sh ble* gene in *E. coli* (EM7 promoter), *S. hindustanus*-derived *ble* gene conferring resistance to Zeocin (Sh ble), and the *S. cerevisiae*-derived *CYC1* transcription termination region (CYC1Sc TT). The pH4 vector (**B**) contains the selection marker expression cassette composed of *C. santamariae* G12-derived *TEF1* promoter (TEF1G12 promoter), synthetic prokaryotic EM7 promoter, *Sh ble* gene conferring resistance to Zeocin, and the *S. cerevisiae*-derived *CYC1* transcription termination region (CYC1Sc TT). The pH5A vector (**C**) contains the selection marker expression cassette, composed of *C. santamariae* G12-derived *TEF1* promoter (TEF1G12 promoter), synthetic prokaryotic EM7 promoter, the modified *Sh ble* gene (Sh ble CpG free), and the *C. santamariae* G12-derived *CYC1* transcription termination region (CYC1G12 TT). The pH6 vector (**D**) contains the selection marker expression cassette composed of *D. macquariensis* D50-derived *IEF2* promoter (IEF2D50 promoter), the EM7 promoter, the *Sh ble* gene, and the *S. cerevisiae*-derived *CYC1* transcription termination region (CYC1Sc TT).

A series of expression vectors for heterologous gene expression in *D. macquariensis* yeast strain D50 was constructed on the basis of the *K. phaffii* pGAPZα B shuttle vector (Invitrogen, Carlsbad, CA, USA; now supplied by Thermo Fisher Scientific, Waltham, MA, USA). For this purpose, a constitutive *K. phaffii*-derived *GAP* (glyceraldehyde-3-phosphate dehydrogenase) promoter was replaced with a *GAP* promoter from *D. macquariensis* D50, and a *K. phaffii*-derived *AOX1* (alcohol oxidase 1) transcription termination sequence was replaced with a *CYC1* (cytochrome C isoform 1) transcription termination region from *D. macquariensis* D50. The expression cassette of each plasmid also contains a sequence encoding a signal peptide (pre-pro-peptide) of the α-mating factor (α-MF) from *S. cerevisiae* with the Kex2 cleavage site, multiple cloning site (MCS), a C-terminal *myc* epitope (EQKLISEEDL), and a polyhistidine tag for western blotting and IMAC (Immobilized Metal Affinity Chromatography) purification of recombinant proteins (Figure 3). The selection of both *D. macquariensis* D50 and *E. coli* transformed with developed plasmids has been provided by the *Sh ble* gene (*Streptoalloteichus hindustanus ble* gene) product conferring resistance to antibiotic Zeocin. In the pH3 vector expression of the *Sh ble* gene has been directed by a constitutive *TEF1* (translation elongation factor 1α) promoter and *CYC1* transcriptional terminator from *S. cerevisiae*, as in the pGAPZα B vector (Figure 4A). In the pH4 vector, the *TEF1* promoter from *S. cerevisiae* was replaced with a *TEF1* promoter from *C. santamariae* G12 (Figure 4B), whereas in the pH6 vector it was replaced with an *IEF2* promoter from *D. macquariensis* D50 (promoter of a gene encoding an isoform of a translation EF-1α of *D*. *macquariensis* D50) (Figure 4D). In the case of pH5A vector, the selection marker cassette contains the *TEF1* promoter from *C. santamariae* G12, an *Sh ble* CpG free gene variant from p-MOD2-Zeo plasmid (InvivoGen, Toulouse, France), and a *CYC1* transcription termination sequence from *C. santamariae* G12 (Figure 4C). Moreover, all constructed vectors contain the pUC origin of replication for propagation in *E. coli*.

### 2.5. Construction of Expression Plasmids with a bgaL Gene Cloned into the Expression Cassette and Generation of Recombinant D. macquariensis D50 Strains

To assess the functionality of developed expression vectors, a *bgaL* gene-encoding cold-active β-d-galactosidase of *Paracoccus* sp. 32d [37] was cloned into pH3, pH4, pH5A, and pH6 plasmids under the control of *D. macquariensis* D50-derived *GAP* promoter, downstream of the sequence-encoding signal peptide of *S. cerevisiae* α-MF. The resulted expression plasmids were digested with *Bsp*HI restriction enzymes within the *GAP* promoter for linearization. Then, the competent cells of *D. macquariensis* D50 were transformed with the linear plasmids according to the developed electroporation method. The number of transformants obtained varied depending on the plasmid used and was 444 for pH3Bgal32d (89 per μg of DNA), 523 for pH4Bgal32d (105 per μg of DNA), 86 for pH5ABgal32d (17 per μg of DNA), and 10 for pH6Bgal32d (2 per μg of DNA). All yeast transformants were Zeocin-resistant, but some of them lacked β-d-galactosidase activity and formed cream-colored colonies on agar medium supplemented with the antibiotic and synthetic β-d-galactosidase substrate, X-gal (5-bromo-4-chloro-3-indolyl β-d-galactopyranoside) (Table 4).

A range of *D. macquariensis* D50 transformants, with and without enzyme activity, were screened by nested PCR for the presence of the *bga*L gene of *Paracoccus* sp. 32d in the genome. Positive-nested PCR results were obtained for all transformants tested. Recombinant strains of *D. macquariensis* D50 were found to be mitotically stable. They showed resistance to the antibiotic and β-d-galactosidase activity during 50 passages on YPD-agar plates supplemented with Zeocin and X-gal. They were also stable when repeatedly passaged on the medium without antibiotics.

### 2.6. Production of Recombinant Paracoccus *sp.* 32d β-d-Galactosidase in the D. macquariensis D50 Expression Host

For the production of the cold-active *Paracoccus* sp. 32d β-d-galactosidase in the *D. macquariensis* D50 expression host, recombinant microorganisms were grown in fermenters in optimized medium and optimized physical conditions. Most of the β-d-galactosidase activity (approx. 98%) remained in yeast cells.

### 2.7. Preparation of Cell-Free Extracts and Purification of the Enzyme

The recombinant *Paracoccus* sp. 32d β-d-galactosidase produced in the *D. macquariensis* strain D50 was mostly accumulated inside yeast cells; therefore, it was necessary to develop an effective method of its extraction from biomass. For this purpose, the following methods of yeast cells disintegration were tested: sonication, enzymatic-chemical lysis using YeastBuster Protein Extraction Reagent (Novagen, Madison, WI, USA; now supplied by Merck KGaA, Darmstadt, Germany), and disintegration using glass beads and lysis buffer. The highest protein concentration in cell-free extract was obtained using the biomass sonication method, however, the enzyme obtained in this way did not show activity due to excessive heating of the sample during the sonication process and probable thermal inactivation of the enzyme. Therefore, in further research, the method of disintegration of yeast biomass using glass beads and lysis buffer was used. This method yielded about 0.6 g of protein with an activity of 1150 U from 1 L of yeast culture (Table 5).

**Figure 5:**
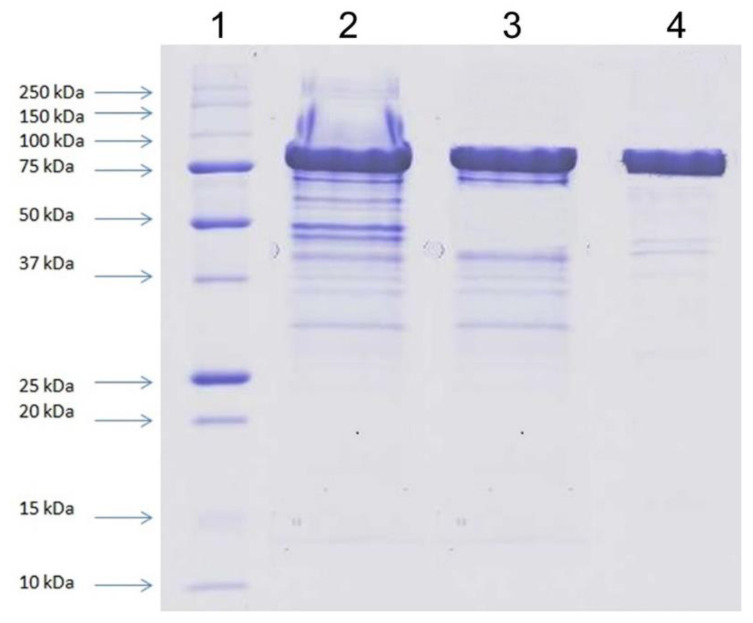
SDS-PAGE protein patterns of fractions obtained after successive purification steps of the recombinant *Paracoccus* sp. 32d β-d-galactosidase produced in the *D. macquariensis* D50 expression host. Lane 1—protein molecular weight marker (Protein precision plus unstained, BioRad, Hercules, CA, USA), lane 2—pooled fraction after ion-exchange chromatography on HiTrap Q FF column, lane 3—pooled fraction after ion-exchange chromatography on Mono Q 5/50 GL column, lane 4—pooled fraction after size exclusion chromatography on Superdex 200 pg column.

The recombinant BgaL enzyme was purified by using the three-step procedure, presented in Table 6. The specific activity of purified *Paracoccus* sp. 32d β-d-galactosidase was approx. 47 U/mg, with a 23 purification fold and 49% of activity recovery. Coomassie-stained SDS polyacrylamide gel of purified enzyme preparation showed one dominant protein band corresponding to the molecular mass of approx. 80 kDa (Figure 5).

Optimal temperature, relative activity at 10 °C, and thermostability of the recombinant *Paracoccus* sp. 32d β-d-galactosidase produced in the *D. macquariensis* D50 expression host were comparable to these properties of the enzyme produced in *E. coli*-based expression system [37] (Table 7). The results obtained for recombinant proteins produced in bacterial and yeast expression hosts prove that the developed expression system based on *D. macquariensis* strain D50 is useful for the production of an active recombinant *Paracoccus* sp. 32d β-d-galactosidase while maintaining the key properties of this hydrolase as an enzyme active at low temperatures [37].

## 3. Discussion

The present study was aimed at creating a novel eukaryotic host/vector system working at low temperatures, which allows the production of recombinant proteins from various origins, including thermolabile proteins from cold-adapted organisms. For this purpose, we looked for a yeast with the following properties among environmental isolates: (1) belonging to the Ascomycota phylum (as well as other yeast expression hosts, namely *S. cerevisiae*, *K. phaffii*, *O. polymorpha*, *Y. lipolytica*, *K. lactis*, *B. adeninivorans*), (2) rapid growth at temperatures below 30 °C (because the decrease in temperature may result in a higher yield of the soluble recombinant protein and is relevant in industrial processes, since it reduces microbial contamination and heating costs), (3) large biomass yield, (4) assimilation of various carbon sources (which make it possible to choose a low-cost substrate), (5) no or weak activity of extracellular proteases (to reduce the risk of degradation of secreted recombinant proteins), and (6) haploidy (for easier genetic manipulation, in the particular rapid selection and higher stability of recombinants after transformation with foreign DNA).

The presence of cold-adapted (psychrophilic and psychrotolerant) yeasts in worldwide cold regions, such as the Arctic, Antarctic, high mountains of Asia, Europe and America, low-temperature deserts, and deep sea, has been extensively documented [39,40,41,42,43,44,45,46,47,48,49]. Most of them belong to the Basidiomycota phylum [39,40,41,42,43,44,45,46,47,48,49]. Concerning Ascomycota, *Debaryomyces hansenii* was reported as a regular inhabitant of cold ecosystems [39,43,45,46,47,48]. The D50 strain isolated from the Antarctic soil sample, which was identified as *D. macquariensis*, best met the project’s objectives and was selected as a promising expression host. It grew optimally at 20 °C and utilized 17 out of 19 tested carbon compounds, including different monosaccharides (pentoses and hexoses), disaccharides, and polyols. For comparison, *D. hansenii* IBT-D1 and *D. macquariensis* IBT-D11 assimilated 16 and 13 of the analyzed carbon sources, respectively [48]. The *D. macquariensis* D50 did not utilize lactose and showed no β-d-galactosidase activity, thus the cold-active β-d-galactosidase of *Paracoccus* sp. 32d [37] could serve as a model protein to test the developed host/vector system.

The genome of *D. macquariensis* strain D50 was found to have six chromosomes with a total size of 11.43 Mb, in contrast to *D. macquariensis* strain IBT-D11 with nine chromosomes with a total size of 20.94 Mb [48]. The results show that the genomes of different strains of the species *D. macquariensis* can differ significantly in the number and size of chromosomes. Similarly, a total size of the genome to *D. macquariensis* D50 revealed *D. hansenii* strain IBT-D1 with six chromosomes with a total size of 12.48 Mb [48]. This species has also many varieties. Some *D. hansenii* strains have been found to contain somewhere between seven and ten chromosomes with a length of 0.31–3.14 Mb [50]. For example, the strains belonging to *D. hansenii* var. *hansenii* have genomes with a size of approx. 12.6 Mb, consisting of six chromosomes with a length of 1.36–3.14 Mb, while *D. hansenii* var. *fabryi* has seven chromosomes (from 0.31 to 2.68 Mb) with a total genome size of 11.9 Mb [50]. The strain type of *D. macquariensis* (CBS 5572) [51], previously classified as *D. hansenii* var. *fabryi*, has a genome with a size of 12.26 Mb consisting of seven chromosomes with a length of 1.08–2.80 Mb [50]. A comparison of genome size obtained by FCM with PFGE results revealed that *D. macquariensis* strain D50 is a haploid, as well as *D. hansenii* strain IBT-D1 [48], the strain type of *D. hansenii* var. *hansenii* (CBS 767) [52], and *D. macquariensis* strain IBT-D11 [48]. Concerning the employed yeast expression hosts, both *S. cerevisiae* and *K. phaffii* are homothallic yeasts that can exist in both haploid and diploid states; however, *K. phaffii* is more stable as a haploid [53]. The *K. lactis* is heterothallic with a predominantly haploid life cycle [22], whereas *B. adeninivorans* is considered to be a haploid asexual yeast [25].

The *D. macquariensis* expression system includes the D50 host strain and four shuttle expression/integration vectors equipped with the *Streptoalloteichus hindustanus*-derived *ble* gene, conferring resistance to Zeocin for the selection of recombinant strains of both *E. coli* (during cloning a gene of interest) and *D. macquariensis* (after transformation with expression plasmid). Dominant selection markers, such as antibiotic resistance genes, are used when auxotrophic yeast strains are not available. The G418 resistance gene is the most popular. It has been employed in expression vectors designed for *S. cerevisiae*, *K. lactis*, *O. polymorpha*, and *K. phaffii* [6,7,20,22]; however, the *D. macquariensis* strain D50 showed natural resistance to G418. The hygromycin B resistance gene serves as the dominant selectable marker in *B. adeninivorans* and *O. polymorpha*-derived expression platforms, and in a wide-range integrative yeast expression vector based on *B. adeninivorans*-derived elements [7,24,54], while the *Sh ble* gene conferring resistance to Zeocin is used in *K. lactis* and *K. phaffii* expression systems [6,7,20,22].

The production of recombinant proteins in yeast cell factories can be done using three types of vectors: episomal plasmids, centromeric plasmids, and integrative plasmids. Episomal plasmids maintained in high-copy number inside the cell enables robust gene expression but are mitotically unstable and are, consequently, not suitable for the development of industrial strains. Centromeric plasmids are more stable but provide limited gene expression due to the low copy number in the cell. Hence, the integrative plasmids are most suitable for genetic manipulation of yeast expression hosts, including *S. cerevisiae*, *K. phaffii*, *O. polymorpha*, *B. adeninivorans*, and others [6,20,21,24,54]. The genomic integration of expression vector allows the removal of selective pressure, for example, an antibiotic, after constructing a recombinant strain. The expression vectors of the pH series contain *D. macquariensis* D50-derived sequences allowing specific insertion into the host genome, namely the 5′ flanking region of the *GAP* gene with a unique restriction site for linearization, because the use of linear plasmid containing terminal regions, which are homologous to sequences in the *D. macquariensis* D50 genome facilitates chromosomal integration.

The central element of any expression vector is an expression cassette containing a promoter and a transcriptional terminator. Promoters are responsible for driving the expression of heterologous genes, while transcriptional terminators serve a mechanistic role and influence mRNA stability. Both inducible and constitutive promoters with strong transcriptional activity are used in yeast systems. *K. phaffii* and *O. polymorpha* expression cassettes are generally constructed using strong inducible alcohol oxidase 1 gene (*AOX1*) and methanol oxidase gene (*MOX*) promoters, respectively [5,7,20,21]. In *S. cerevisiae*, the galactose-induced *GAL1* promoter is frequently used [6,7]. The same promoter is also used in the *K. lactis* expression system, confirming the transferability of this genetic element across different yeast species [5,6,22]. Inducible promoters are commonly used when separation of growth and production is desired, for example in the production of toxic proteins. However, strong constitutive promoters are also frequently employed to direct high-level expression of heterologous genes in various yeast species, including *S. cerevisiae*, *K. phaffii*, *O. polymorpha*, *Y. lipolytica*, and *B. adeninivorans*. Some *K. phaffii* and *O. polymorpha*-based platforms utilize endogenous promoters of glyceraldehyde-3-phosphate dehydrogenase genes (*GAP*) instead of methanol-inducible ones [5,6,7,20,21]. The endogenous promoters of translation elongation factor 1α genes (*TEF1*) have been used in *S. cerevisiae*, *Y. lipolytica*, *B. adeninivorans*, and *K. phaffii* expression systems [5,6,20,24]. In addition, it has been demonstrated that the *B. adeninivorans*-derived *TEF1* promoter is active in *S. cerevisiae*, *O. polymorpha, K. phaffii, D. hansenii*, and *D. polymorphus* [54,55]. Constitutive promoters offer simplicity, relatively constant levels of expression, and are active regardless of the carbon source used in the medium; therefore, such promoters were selected for the development of the *D. macquariensis* D50 host/vector system. During the research, the transcriptional activity of endogenous *GAP* and *IEF2* putative promoters, and the heterologous *C. santamariae* G12-derived *TEF1* putative promoter was confirmed, and they were used to construct a series of pH expression vectors for *D. macquariensis* D50 host strain. On the other hand, the putative promoter of the *D. macquariensis* D50 *TEF1* gene turned out to be inactive. The *D. macquariensis* D50 yeast cells transformed with plasmid DNA carrying the *Sh ble* gene under the control of homologous *TEF1* promoter exhibited β-d-galactosidase activity but were unable to grow on the medium supplemented with Zeocin. In this case, the *bga*L gene of *Paracoccus* sp. 32d was used as a selection marker and the *D. macquariensis* D50 cells immediately after transformation were plated on an agar medium containing only X-gal. In this way, without an antibiotic selective pressure, only two recombinants were obtained. According to the literature, the presence of multiple genes for EF-1α in eukaryotes or EF-Tu in bacteria is a common feature. The *S. cerevisiae* haploid genome contains two genes coding for the translation elongation factor 1α, called *TEF1* and *TEF2*, and both genes are strongly transcribed [56,57]. Similar results were obtained for *Candida albicans*, which was able to produce mRNA from both genes with no apparent quantitative differences. The coding regions of *C. albicans TEF1* and *TEF2* differed by only five nucleotides and encoded identical proteins, but 5′- and 3′-flanking regions were not homologous [58]. In other cases, multiple genes are regulated differently, for example, three genes for EF-1α in *Mucor racemosus* [59] or two genes for EF-Tu in *E. coli* [60]. In contrast, the *B. adeninivorans* has only one gene-encoding translation elongation factor, 1α [61]. Thus, the *TEF1* gene in *D. macquariensis* D50 may be transcriptionally inactive or otherwise regulated than *IEF2*.

With regard to transcriptional terminators, endogenous *AOX1* and *MOX* terminators are commonly used in *K. phaffii* and *O. polymorpha* expression cassettes, respectively. Most often, used *S. cerevisiae* expression vectors contain endogenous *CYC1* or *ADH1* terminators. The *S. cerevisiae*-derived transcriptional terminators have also been used in other yeast expression systems, namely *K. lactis*, *K. phaffii*, *O. polymorpha*, and *Y. lipolytica* [5]. The functionality of *S. cerevisiae*-derived terminators across non-conventional yeast hosts suggested their utility in the novel expression system. In our research, we confirmed the functionality of the endogenous putative *CYC1* transcriptional terminator, as well as the heterologous *CYC1* terminator derived from *S. cerevisiae* and the putative *CYC1* terminator from *C. santamariae* G12 in the *D. macquariensis* D50 expression host. The *K. phaffii*-derived *AOX1* terminator, as part of the pH1 expression vector, was also active in *D. macquariensis* D50 cells. As a result of the transformation of *D. macquariensis* D50 cells with the linear form of the pH1Bgal32d plasmid containing the *bga*L gene of *Paracoccus* sp. 32d under the control of homologous *GAP* promoter and the *K. phaffii*-derived *AOX1* transcriptional terminator, 14 recombinants with the Zeo^R^, β-gal^+^ phenotype, and 16 recombinants with the Zeo^R^, β-gal^−^ phenotype were obtained. The overall transformation efficiency was 6 per μg of DNA.

Recombinant proteins can accumulate in the cytoplasm or they can be secreted into the culture medium, but signal sequences are required for protein secretion in yeasts. Cytoplasmic accumulation often achieves high yields, but additional steps are required for protein purification. In contrast, the secretion of recombinant proteins into the culture medium allows for simple and cost-effective purification. The native signal peptide is often sufficient for secretion when present on a recombinant protein. For example, the recombinant human serum albumin (rHSA) was efficiently secreted into the culture medium by *B. adeninivorans* [24], *K. lactis* [62], and *O. polymorpha* [63]. Several endogenous and heterologous signal sequences have also been used to secrete proteins in yeast expression hosts [6,20,22,23]. Due to the fact that the conservation among eukaryotic signal sequences is strong, the pre-pro-sequence of the *S. cerevisiae* α-mating factor (α-MF) is commonly used for recombinant proteins secretion in non-*Saccharomyces* yeasts, e.g., *K. lactis* [6,22], *B. adeninivorans* [55], *O. polymorpha* [55,64,65], and *K. phaffii* [6,20,66,67,68]. Moreover, the signal sequence usually comprises a Kex2 protease recognition site, which is located between the signal peptide and the heterologous protein for proteolytic maturation. For this reason, the pre-pro-sequence of the *S. cerevisiae* α-MF was used for the secretion of *Paracoccus* sp. 32d β-d-galactosidase in *D. macquariensis* D50, however, it did not have the expected effect. Since the *D. macquariensis* D50 naturally secretes little protein into the medium, it may have limited secretion capacity, like *S. cerevisiae*, but this requires further investigation.

As mentioned before, the integration of linear plasmid DNA carrying the heterologous gene into the host chromosome provides genetically stable expression strains. This requires the action of a double-stranded break (DSB) repair mechanism by homologous recombination (HR) or non-homologous end-joining (NHEJ). Both pathways are highly conserved and coexist in most cells, but one of them can be preferred. *S. cerevisiae* strongly favors HR, whereas non-conventional yeasts, such as *Y. lipolytica*, *K. phaffii*, *K. lactis*, *O. polymorpha*, and *B. adeninivorans*, show a stronger preference for NHEJ. Non-homologous end-joining results in random integration of all or part of a vector into the genome instead of highly specific integration provided by homologous recombination. In *S. cerevisiae*, short overhangs of about 50 bp flanking the expression cassettes are sufficient to achieve almost exclusively specific integration, whereas in *K. phaffii* even long overhangs (~1 kb) may result in only <1 to 30% correct integration [5,69,70,71]. In *D. macquariensis* D50, medium-length flanking arms homologous to the genome in the *GAP* promoter locus (286 and 214 bp) resulted in two major subpopulations of transformants, namely with and without β-d-galactosidase activity. To resolve the problem with nonspecific integration, *Y. lipolytica* and *K. phaffii* strains with *KU70* genes (encoding NHEJ-promoting proteins) deletions have been constructed [70,72]. Moreover, it has been shown that hydroxyurea (HU) treatment prior to transformation enriches S-phase cells that are highly active in homologous recombination and thus increase the gene-targeting efficiency in non-conventional yeasts [69]. Unfortunately, this method was not successful in the case of *D. macquariensis* D50. Following the procedure described by Tsakraklides et al. [69], we did not obtain any *D. macquariensis* D50 transformants.

In conclusion, the described expression system allowed, for the first time, the production of a recombinant protein in the cold-adapted eukaryotic host. However, the *D. macquariensis* D50 cell factory is still in a very early stage of development, and further improvements are required, in particular, to increase the yield and secretion of proteins. Despite these drawbacks, the *D. macquariensis* D50 expression system can significantly contribute to research on various proteins, especially those that are difficult to express and require post-translational modifications. Therefore, it will also be tested in terms of PTMs of recombinant proteins, in particular, the glycosylation pattern.

## 4. Materials and Methods

### 4.1. D50 Strain Isolation and Taxonomic Identification

The yeast strain D50 was isolated from a soil sample collected in the vicinity of Henryk Arctowski Polish Antarctic Station on King George Island and more specifically in the vicinity of penguin ridge—path to the summit, a place covered with grass (62°08′44.6″ S, 58°27′43.1″ W). The sample was stored in the sterile plastic tube at 4 °C until analysis. The yeast isolation procedure has been described previously by Białkowska et al. [48]. The strain was stored in the form of glycerol stocks (1:1) at −80 °C. Cryogenic culture of D50 strain was also prepared using Roti^®^-Store yeast cryo-vials with the special freezing medium (Carl Roth GmbH Co. KG (Karlsruhe, Germany).

Genetic identification of strain D50 was conducted by PCR amplification of the D1/D2 domains of the large subunit rRNA gene region and the internal transcribed spacers (ITS1, 5.8S rRNA gene and ITS2 regions) according to Turchetti et al. [73]. The amplified fragments were sequenced by the company Genomed S.A. (Warsaw, Poland). The sequences were aligned, analyzed, and corrected using Nucleic Acid Sequence Massager software (http://www.cmbn.no/tonjum/seqMassagersaf.htm, accessed on 12 May 2015) and compared with sequences from the international GenBank database (http://www.ncbi.nlm.nih.gov, accessed on 12 May 2015) using a nucleotide BLAST search tool.

### 4.2. Determination of the Size of the Genome and Ploidy of the D50 Strain

Flow cytometry (FCM) analysis and pulsed-field gel electrophoresis (PFGE) of genomic DNA were used for determining the size of genome, ploidy, and electrophoretic karyotype of the D50 strain. Ploidy of yeast strain was calculated from FCM/PFGE results. Detailed procedures used in FCM (cell preparation and DNA staining; flow cytometry analysis) and PFGE (immobilization of yeast DNA in agarose plugs; pulsed-field gel electrophoresis) have been described previously by Białkowska et al. [48].

### 4.3. Biochemical and Physiological Characterization of the D50 Strain

The yeast D50 strain was tested for its ability to grow at 0, 4, 10, 15, 20, 30, and 37 °C on YPD agar plates (1% yeast extract, 2% bactopeptone, 2% glucose, 1.5% agar) and in liquid YPD (content as above without agar) at 150 rpm. Yeast growth was monitored over 7 days. The ability of the D50 strain to utilize different carbon sources, its enzymatic activity and sugar fermentation ability were evaluated using API 20 C AUX, API ZYM, and API Candida tests (bioMerieux, Marcy-l’Étoile, France), respectively, at 20 °C. Analyses were conducted following the manufacturer’s instructions. Nitrogen sources assimilation tests were performed according to the Lab Manual for Yeast Study by Suh S.O., Zhang N., Nguyen N., Gross S., and Blackwell M. (Mycology Lab Louisiana State University; http://deskuenvis.nic.in/pdf/manual_for_yeast_work_sept2008.pdf, accessed on 12 May 2015). In the first stage, the yeast strain D50 was grown in the 1.17% (*w*/*v*) Yeast Carbon Base medium pH 6.0 for 7 days at 20 °C for nitrogen starvation. Then, three drops of suspension were plated onto YCB-agar plate used as a control (no nitrogen source) and YCB-agar plates, supplemented with ammonium sulfate (400 μg/mL), potassium nitrate (300 μg/mL), sodium nitrite (420 μg/mL), ethylamine hydrochloride (260 μg/mL), l-lysine (660 μg/mL), cadaverine dihydrochloride (540 μg/mL), creatine (400 μg/mL), creatinine monohydrate (340 μg/mL), d-glucosamine (660 μg/mL), imidazole (200 μg/mL), and d-tryptophan (640 μg/mL). Inoculated plates were incubated at 20 °C for 14 days. Yeast growth on various media was observed and compared after 7 and 14 days. Yeast Carbon Base, ethylamine hydrochloride, l-lysine, d-tryptophan, cadaverine dihydrochloride, creatine, and d-glucosamine were purchased from Sigma-Aldrich (Saint Louis, MO, USA). Ammonium sulfate, potassium nitrate, and sodium nitrite were purchased from POCH (Gliwice, Poland). Creatinine monohydrate and imidazole were purchased from AppliChem (Darmstadt, Germany). The proteolytic activity of the D50 strain was studied in two ways: (1) by examining the formation of halos around colonies growing on plates with YPD agar medium supplemented with skimmed milk (2%) at 20 °C; and (2) by assaying the extracellular proteolytic activity of the strain according to Anson [74] with urea-denatured hemoglobin as a substrate after 48 and 72 h cultivation of D50 strain at 20 °C in liquid YPD medium, supplemented with casein (0.5%). The activity was expressed as nanomoles of l-tyrosine released from the substrate per minute under standard conditions (20 °C, 30 min, assays were taken at pH 4.0 and 7.0). The salinity tolerance of the D50 strain was evaluated on YPD agar plates supplemented with 10, 15, and 20% NaCl. The sensitivity of the D50 strain to antibiotics Zeocin and G418 was tested on YPD agar plates supplemented with 25, 50, 100, 200, and 300 μg/mL of Zeocin (Invitrogen, Carlsbad, CA, USA; now supplied by Thermo Fisher Scientific, USA) or 50, 100, 300, 500, 750, and 1000 μg/mL of G418 disulfate salt (Sigma-Aldrich, Saint Louis, MO, USA). The yeast was grown for 7 days at 20 °C.

### 4.4. Optimization of the D50 Strain Growth

For the selection of the most optimal medium for the growth of wild *D. macquariensis* strain D50 the cultures were carried out in microplates in a volume of 1.5 mL in a BioLector microbioreactor at 20 °C and 800 rpm. As a source of carbon, we tested glucose, glycerol and sucrose. As nitrogen sources, we tested corn soak, yeast extract, malt extract, ammonium hydrogen phosphate, and ammonium sulfate. The initial range tested for carbon sources was 0.5–13% and for nitrogen sources 0.25–3.0%.

The physical culture conditions for the wild *D. macquariensis* strain D50 were optimized using Taguchi methodology [75] to maximize the growth of this yeast strain. The optimized factors included the temperature, pH, aeration, and stirring of the yeast culture at 3 levels (Table 8). The L_9_ orthogonal array consisted of 9 experimental runs testing 3 levels of 4 factors, which is equivalent to 3^4^ setups (Table 9). The signal-to-noise ratio (*S*/*N*, η) was calculated from experimental data using the higher-the-better function according to the formula:

(1) $$\left(\frac{S}{N}\right)=-10\log_{10}\left[\frac{1}{n}\times \sum {\left(\frac{1}{y_{i}}\right)}^{2}\right]$$

where *y_i_* is the *i*th quality parameter and *n* is the number of trials.

(2) $${\left(\frac{S}{N}\right)}_{optimal\;for\;the\;process}={\left(\frac{S}{N}\right)}_{process}+\sum \left|{\left(\frac{S}{N}\right)}_{significant\;value}-{\left(\frac{S}{N}\right)}_{process}\right|$$

The process parameters, which had a significant impact on biomass production were investigated by analysis of variance (ANOVA) in the Minitab 18 software. All batch cultures of *D. macquariensis* strain D50 were conducted for 75 h under aerobic conditions in 0.75 L Sixfors fermenters (Infors, Basel, Switzerland) at loading rates of 23%, in the medium composed of glucose (5%) and yeast extract (2%). An antifoam agent used in these processes was AntiFoam 204 (Sigma-Aldrich, Saint Louis, MO, USA). Samples of culture broth were collected every 12 h, and biomass density and pH were measured.

The recombinant *D. macquariensis* strain D50 was cultivated in the conditions the same as for the wild D50 strain. The medium was modified, and to increase the expression of *Paracoccus* sp. 32d β-d-galactosidase gene, it was added 1% peptone tryptone (BTL, Lodz, Poland).

### 4.5. Sequencing and Bioinformatic Analyses of the D. macquariensis Strain D50 Genome

The *D. macquariensis* D50 genomic DNA, extracted with ExtractMe DNA Yeast kit (Blirt S.A., Gdansk, Poland), was used to construct two kinds of genomic libraries. The first library, which contained “short” fragments of genomic DNA (~400 bp) was constructed using NEBNext^®^DNA Library Prep Master Mix Set for Illumina^®^ (New England Biolabs, Ipswich, MA, USA). The second and third libraries, which contained “long” genomic DNA fragments (~5 kbp and ~10 kbp), were constructed with Nextera Mate Pair Library Prep Kit (Illumina, San Diego, CA, USA). The libraries were sequenced with MiSeq (Illumina, San Diego, CA, USA) at Genomed S.A. (Warsaw, Poland). The raw sequencing data were assembled into contigs with CLC Genomic Workbench v.7.0 (QIAGEN Bioinformatics, Hilden, Germany), and the resulted contigs were assembled into scaffolds using SSPACE-BASIC 2.0 [76]. Next, the gene prediction and annotation were done with GeneMark-ES version 2 [77], Blast2GO [78], and InterProScan [79], and the results were combined and analysed with the use of the bioinformatic scripts designed, developed, and tested by Genomed S.A. (Warsaw, Poland). Moreover, the tRNA genes prediction was done with tRNAscan-SE v. 2.0 [80].

For manual annotation, the predicted DNA sequences of *TEF1*, *IEF2* (paralog of *TEF1* gene), *CYC1*, and *GAP* genes of *D. macquariensis* strain D50, and the amino acid sequences of their expression products (in silico translation) were searched against the appropriate NCBI databases with appropriate BLAST tools. Next, the DNA sequences upstream confirmed *TEF1*, *IEF2*, and *GAP* genes of *D. macquariensis* strain D50 were searched for prediction putative TATA-box or TATA-like elements in core promoter DNA sequences with use of YAPP on-line tool (http://www.bioinformatics.org/yapp/cgi-bin/yapp.cgi, accessed on 12 May 2015) [81]. We choose this bioinformatic tool after comparing the location and DNA sequences of predicted YAPP TATA-box or TATA-like elements with the location and DNA sequences of existing TATA-box or TATA-like elements in the promoters of *TEF1* gene of *B. adeninivorans* (GenBank Accession Number: Z47379, [61]), *S. cerevisiae TEF1* gene promoter [82], and the promoters of other *S. cerevisiae* genes, i.e., *ARG1*, *ADH2*, *GCY1*, *HSC82*, *LEU2*, *HIS4*, *HIS3*, *CLN2*, *INO1*, *SUC2*, reported and deposited in the Promoter Database of *S. cerevisiae* (SPPD database, http://rulai.cshl.edu/SCPD/, accessed on 12 May 2015). For the promoters of listed genes, except for one analysed case, i.e., ARG1 gene promoter, the predicted TATA-box or TATA-like sequences with the highest score (>cutoff score, default 0.80) cover the existing analogous sequences in analysed promoter sequences. Then, the results of the TATA-box or TATA-like sequences predictions for above listed *D. macquariensis* strain D50 genes were compared and assessed with respect to the knowledge that (1) typical eukaryotic core promoter lengths of 100–200 bp [83], (2) the location of TATA box in *S. cerevisiae* yeast promoter was found located in a wide range of 40–120 bp upstream of TSS [83], (3) the location of TATA box in other yeast *Schizosaccharomyces pombe* promoter was found located in a range 25–40 bp of TSS (the more typical eukaryotic mode of transcription initiation than in *S. cerevisiae*) [84], and (4) the mean of 5′ UTR lengths is remarkably similar across diverse taxonomic classes, ranging only from ∼100 to ∼200 bp, for example, for *S. cerevisiae* and *C. albicans* yeasts, the mean lengths for 5′UTR are 96.5 bp and 120.9 bp, respectively [85]. Due to the lack of TATA box consensus sequence in *S. cerevisiae TEF1* core promoter replaced by TATA-like sequence AATAAAAA [82], and publicized data that only ~20% of *S. cerevisiae* promoters contains a canonical TATA box [86], we assumed that we would treat each putative TATA-like elements equivalent to the putative TATA box elements when analyzing the prediction results obtained using YAPP tool. On this base, we estimated the length of DNA sequences located upstream, *TEF1*, *IEF2*, and *GAP* genes of *D. macquariensis* strain D50, which may contain their putative promoter regions.

Next, the selected DNA fragments of *D. macquariensis* strain D50 genome were amplified by PCR, sequenced (Genomed S.A., Warsaw, Poland), and analysed (DNA sequencing results of PCR products were aligned with appropriate DNA sequences of *D. macquariensis* strain D50 genome, previously analysed with YAPP on-line tool). After that, all putative promoter sequences were used for construction of pH expression vectors. The manual annotation of the draft genome sequence of *D. macquariensis* strain D50 also confirmed the presence of the *CYC1* gene and the 3′ end of this gene; the putative transcriptional terminator was also used for the construction of pH expression vectors.

### 4.6. Construction of Expression Vectors and Plasmids with the bgaL Gene

*E. coli* TOP10 (Invitrogen, Carlsbad, CA, USA; now supplied by Thermo Fisher Scientific, USA) served as a host strain for cloning and plasmid propagation. Recombinant *E. coli* strains were grown in low salt LB medium pH 7.5 (1% peptone K, 0.5% yeast extract, 0.5% NaCl), supplemented with Zeocin (25 μg/mL) at 37 °C, with shaking at 180 rpm. Agar plates were prepared by adding 1.5% bacteriological agar to the medium.

Table 10 summarizes oligonucleotide primers and PCR products used in this study.

#### 4.6.1. Construction of the pH3 Expression Vector

The first stage leading to the construction of the pH3 expression vector was obtaining a PCR product designated as H3.1 and containing a sequence of the *CYC1* transcriptional terminator from *D. macquariensis* D50 in order to clone it in the pGAPZα B vector (Invitrogen, Carlsbad, CA, USA). The PCR was performed using a genomic DNA of *D. macquariensis* D50 as a template and primers designated as CYCD50zaAOX1for and CYCD50zaAOX1rev (Table 10). The obtained PCR product of 312 bp was purified using an Extractme DNA Clean-Up Kit (Blirt S.A., Gdansk, Poland). The purified PCR product and pGAPZα B plasmid were digested with *Bam*HI and *Age*I restriction enzymes (Thermo Fisher Scientific Baltics UAB, Vilnius, Lithuania), followed by purification via DNA precipitation. The purified DNA fragments were then combined using T4 DNA ligase purchased from Epicentre Biotechnologies (Madison, WI, USA). The obtained ligation mixture was used to transform *E. coli* TOP10 cells (Invitrogen, Carlsbad, CA, USA), and recombinant plasmids were then isolated using an Extractme Plasmid DNA Kit (Blirt S.A., Gdansk, Poland). The second stage, leading to construction of the pH3 expression vector, involved obtaining a PCR product designated as H3.2 and containing a sequence of the *GAP* promoter from *D. macquariensis* D50. A PCR was performed with a genomic DNA of *D. macquariensis* D50 as the template and primers designated as pGsTD50_forward pGsTD50_reverse (Table 10). The obtained PCR product of 518 bp was then purified and cloned into the pGAPzCYCD50bezAOX1 plasmid, at *Bgl*II and *Bst*BI restriction sites. The correct construction of the pH3 expression vector was confirmed by DNA sequencing (Genomed S.A., Warsaw, Poland).

#### 4.6.2. Construction of the pH4 Expression Vector

For the construction of the pH4 expression vector, a PCR product containing a sequence of the *TEF1* promoter from the psychrotolerant yeast *C. santamariae* strain G12 was obtained and cloned into the pH3 expression vector. The PCR was carried out with a genomic DNA of *C. santamariae* G12 as the template and primers pTEF1pH4_forward and pTEF1pH4_reverse (Table 10). The obtained PCR product designated as H4 of 370 bp was purified using an Extractme DNA Clean-Up Kit (Blirt S.A., Gdansk, Poland). The purified PCR product and DNA of the pH3 expression vector were digested with *Bam*HI and *Vsp*I (*Ase*I) restriction enzymes (Thermo Fisher Scientific Baltics UAB, Vilnius, Lithuania), purified by DNA precipitation, and combined in a DNA ligation reaction. Next, the *E. coli* TOP10 cells (Invitrogen, Carlsbad, CA, USA) were transformed with the ligation mixture, and recombinant plasmids were isolated using an Extractme Plasmid DNA Kit (Blirt S.A., Gdansk, Poland). The correct construction of the pH4 expression vector was confirmed by DNA sequencing (Genomed S.A., Warsaw, Poland).

#### 4.6.3. Construction of the pH5A Expression Vector

The first stage leading to the construction of the pH5A expression vector was obtaining a PCR product designated as H4.1 and containing the sequence of the *Sh ble* CpG free gene, in order to clone it into the pH4 expression vector. For this purpose, the PCR was carried out with a DNA template of the p-MOD2-Zeo plasmid (InvivoGen, Toulouse, France) and primers BleoRCpGfree_For and BleoRCpGfree_Rev (Table 10). The PCR product of 390 bp was purified, digested with *Nco*I and *Bse*RI restriction enzymes (Thermo Fisher Scientific Baltics UAB, Vilnius, Lithuania), and cloned into the pH4 expression vector, which was digested with the same restriction enzymes. This led to the indirect DNA construct, i.e., a plasmid designated as pH4A. The second stage, leading to construction of the pH5A expression vector, involved obtaining a PCR product designated as H5.1 and containing a sequence of the *CYC1* transcriptional terminator from *C. santamariae* G12 in order to clone it into the pH4A plasmid. The PCR was performed with using a genomic DNA of *C. santamariae* G12 as the template and primers CYC1G12_For and CYC1G12_Rev (Table 10), enabling cloning of the PCR product into the pH4A plasmid at *Eco*RV and *Pci*I restriction sites. The obtained PCR product of 588 bp and DNA of the pH4A plasmid were digested with *Eco*RV and *Psc*I (*Pci*I) restriction enzymes (Thermo Fisher Scientific Baltics UAB, Vilnius, Lithuania) and purified by DNA precipitation. The purified DNA fragments were then combined in a DNA ligation reaction, and the ligation mixture was used to transform *E. coli* TOP10 cells (Invitrogen, Carlsbad, CA, USA). The correct construction of the pH5A expression vector isolated from *E. coli* cells was initially verified through restriction analysis and finally confirmed by DNA sequencing (Genomed S.A., Warsaw, Poland).

#### 4.6.4. Construction of the pH6 Expression Vector

The pH6 expression vector was constructed by cloning a PCR product designated as H6 and containing a sequence of the *IEF2* promoter from *D. macquariensis* D50 into the pH3 expression vector. The PCR was performed using a genomic DNA of *D. macquariensis* D50 as the template and primers designated as pIEF2pH6_forward and pIEF2pH6_reverse (Table 10). The obtained PCR product of 684 bp was purified using an Extractme DNA Clean-Up Kit (Blirt S.A., Gdansk, Poland). Next, the purified PCR product and the pH3 vector were digested with *Bam*HI and *Vsp*I (*Ase*I) restriction enzymes (Thermo Fisher Scientific Baltics UAB, Vilnius, Lithuania), purified through DNA precipitation, and ligated. The correct construction of the pH6 expression vector was confirmed by DNA sequencing (Genomed S.A., Warsaw, Poland).

#### 4.6.5. Construction of pH3Bgal32d, pH4Bgal32d, pH5ABgal32d, and pH6Bgal32d Expression Plasmids

The *bgaL* gene encoding β-d-galactosidase of *Paracoccus* sp. 32d was amplified using forward primer F32dBgalXho and reverse primers R32dBgalXba or R32dBgalNot (Table 10). The PCR was performed using DNA polymerase *Hypernova* (Blirt S.A., Gdansk, Poland) and a genomic DNA of *Paracoccus* sp. 32d as a template [37]. The PCR product designated as H346_BGal32d of 2222 bp was purified from the reaction mixture, digested with *Xho*I and *Xba*I endonucleases (Thermo Fisher Scientific Baltics UAB, Vilnius, Lithuania), and cloned into pH3, pH4, and pH6 vectors, digested with the same restriction enzymes. The PCR product designated as H5A_BGal32d of 2223 bp was purified, digested with *Xho*I and *Not*I restriction enzymes (Thermo Fisher Scientific Baltics UAB, Vilnius, Lithuania), and cloned into pH5A vector digested with the same restriction endonucleases. The resulting pH3Bgal32d, pH4Bgal32d, pH5ABgal32d, and pH6Bgal32d recombinant plasmids contained the *Paracoccus* sp. 32d β-d-galactosidase gene under the control of the *GAP* promoter from *D. macquariensis* D50.

### 4.7. Transformation

The pH3Bgal32d, pH4Bgal32d, pH5ABgal32d, and pH6Bgal32d expression plasmids were linearized by digestion with *Bsp*HI restriction enzyme (New England Biolabs, Ipswich, MA, USA), within the *D. macquariensis* D50-derived *GAP* promoter, and then purified through precipitation. Linear, purified DNA of the expression plasmids was concentrated to a concentration of 1 µg/µL and used in electroporation of competent yeast cells of *D. macquariensis* D50.

In order to obtain competent cells, a single colony of *D*. *macquariensis* D50 yeast was used to inoculate 10 mL of the YPD medium (2% peptone K, 1% yeast extract, 2% glucose). The culture was maintained for 22–24 h at 25 °C, with shaking (180 rpm). Next, a 30 µL sample of yeast culture in a stationary phase was collected and used to inoculate 200 mL of sterile YPD medium. The culture was then maintained at 25 °C with shaking (180 rpm) for 12.5–13.0 h until the logarithmic culture growth phase was achieved at a concentration of 5 × 10^7^ cells/mL (OD_600_ = 1). The culture was centrifuged for 4 min at 1500× *g* at room temperature. The supernatant was carefully decanted and the yeast cell pellet was washed twice with 100 mL of sterile, deionized water. The pellet was resuspended in 20 mL of a solution containing 35 mM dithiothreitol and 100 mM lithium acetate, followed by incubation for 45 min to 1 h at 25 °C with gently shaking (80 rpm). After this time, yeast cells were centrifuged for 4 min at 1500× *g* at 4 °C. From this point, the cells and all used reagents were stored at a temperature near 0 °C. The pellet was washed once with 20 mL of sterile, deionized water, followed by washing twice using 20 mL of 1 M sorbitol, with a 4 min centrifugation at 1500× *g* at 4 °C after every stage. Ultimately the pellet was resuspended in 1 M sorbitol for the final volume of 500 µL, and the cells were divided into 10 tubes of 50 µL each.

In order to perform the electroporation, 50 µL of competent *D*. *macquariensis* D50 cells were mixed with 5 µL DNA (concentration 1 µg/µL), suspended in sterile, deionized water and gently mixed with a pipette, followed by incubation in ice, at ca. 0 °C, for 10 min. The sample was then transferred to an ice-cold 0.2 cm electroporation cuvette. Then, the cuvette was placed in a Gene Pulser Xcell^TM^ Electroporation System (Bio-Rad, Hercules, CA, USA) and a pulse of 1.8 kV was applied for 5 ms. 1 mL of a solution made of 1 M sorbitol and YPD medium mixed at a 1:1 ratio was added to the cuvette immediately after the pulse. The yeast cells were then incubated at room temperature for 20 min, without shaking. After this time, the cuvette content was transferred to a 50 mL sterile tube and mixed with 10 mL of the medium containing 5% glucose (2% peptone K, 1% yeast extract, 5% glucose) and incubated at 25 °C for 18–20 h, with shaking (180 rpm). After this time, cells were centrifuged for 10 min at 963× *g* in order to reduce the sample volume to 1 mL, followed by spreading on the YPDS (2% peptone K, 1% yeast extract, 2% glucose, 2% agar, 1 M sorbitol) plates containing Zeocin (25 µg/mL) and X-Gal (5-bromo-4-chloro-3-indolyl β-d-galactopyranoside, 20 µg/mL). After 7 days of incubation at 25 °C, plates were analyzed for transformants colony staining.

### 4.8. Verification of Transformants by Nested PCR

Genomic DNA of *D. macquariensis* D50 transformants was isolated using ExtractMe DNA Yeast kit (Blirt S.A., Gdansk, Poland), according to the manufacturer’s instruction. In the first round of nested PCR, F32dBgalXho and R32dBgalXba primers (Table 10) and PCR Mix Plus HGC (A&A Biotechnology, Gdynia, Poland) were used to amplify the entire *Paracoccus* sp. 32d β-d-galactosidase gene. A second PCR was performed on the products of the first PCR with 2F32dNested and R32dNested primers (Table 10) to amplify an internal sequence of the *bgaL* gene (963 bp).

### 4.9. Production of Recombinant Paracoccus *sp.* 32d β-d-Galactosidase in Batch Culture in Fermenters

The batch cultivation of recombinant *D. macquariensis* D50/pH4Bgal32d was carried out in a 0.75 L Sixfors fermenter (Infors, Basel, Switzerland) in a medium containing 5% of glucose (Chempur, Piekary Slaskie, Poland), 2% of yeast extract (BTL, Lodz, Poland), and 1% of peptone tryptone (BTL, Lodz, Poland) at loading rate of 40%. The initial medium pH was adjusted to 5.5. This process was conducted at 20 °C, aeration of 1.5 vvm, and agitation rate of 300 rpm, for 120 h.

### 4.10. Preparation of Cell-Free Extract

Cell-free extracts from yeast cells harvested on completion of the culture carried out at 20 °C in the liquid medium were prepared by using three different methods: (1) 2 g of the wet biomass was sonicated for 5 min at 0 °C, vibrations amplitude 30% (Vibra Cell 71408, Bioblock Scientific, USA) in 50 mM potassium phosphate buffer, pH 7.6, enriched with 100 mM MgCl_2_, 1 mM PMSF, and 1 mM β-mercaptoethanol. The residual insoluble cell debris was discarded after centrifugation (5000× *g*, 4 °C, 30 min); (2) the enzymatic-chemical lysis of biomass was performed using the commercial YeastBuster Protein Extraction Reagent (Novagen, Madison, WI, USA; now supplied by Merck KgaA, Darmstadt, Germany) according to the manufacturer’s recommendations; (3) 200 mg of glass beads (ϕ 0.25–0.5 mm) were added to 200 mg of cell pellets and were suspended in 1 mL of lysis buffer (20 mM potassium phosphate buffer, pH 7.4, enriched with 0.1% Triton X-100, 100 mM KCl, 8 mM MgCl_2_, 150 mM NaCl, and 1 mM PMSF). The mixture was incubated alternately on a shaker (13,000 rpm at room temperature) and on ice for 30 s each, repeating 12 times. The residual insoluble cell debris was discarded after centrifugation (1500× *g*, 10 °C, 5 min).

### 4.11. Purification and Some Properties of Recombinant Paracoccus *sp.* 32d β-d-Galactosidase

All purification steps were carried out at 10 °C using the liquid chromatography system ÄKTA Basic (GE Healthcare Bio-Sciences, Piscataway, NJ, USA). The cell-free extract obtained from 150 g of wet biomass of yeasts by the disintegration of yeast cells using glass beads and lysis buffer was applied on HiTrap Q FF (5 mL) column previously equilibrated with 50 mM potassium phosphate buffer, pH 6.3. Elution was carried out with a linear NaCl gradient (0–0.5 M) in the starting buffer and with a flow rate of 2 mL/min. β-d-Galactosidase-containing fractions, eluted within the range 0–0.15 M NaCl, were collected and desalted by dialysis. The sample was then loaded to Mono Q 5/50 GL column previously equilibrated with 50 mM potassium phosphate buffer, pH 6.3. Elution was carried out with a linear NaCl gradient (0–0.15 M) in the starting buffer, and with a flow rate of 1 mL/min. The enzyme-containing fractions were pooled, concentrated on a 30 kDa cut-off filter and purified by size exclusion chromatography on Superdex 200 pg 16/600 (flow rate of 1 mL/min), previously equilibrated with 50 mM potassium phosphate buffer, pH 6.3, enriched with 0.15 M NaCl. Purification of the recombinant protein was confirmed by SDS-PAGE analysis.

The optimal temperature of purified β-d-galactosidase was determined by performing the standard activity assay in the range of 0–70 °C with *o*-nitrophenyl β-d-galactopyranoside (ONPG; Sigma-Aldrich, Saint Louis, MO, USA). Thermal stability was measured under standard reaction condition (pH 7.5, 40 °C) after enzyme preincubation without substrate for up to 180 min in temperature range of 4–60 °C, prior to the enzyme activity assay.

### 4.12. Other Analytical Methods

The β-d-galactosidase activity in hydrolysis of ONPG (4 mM) was estimated in 20 mM potassium phosphate buffer, pH 7.5. After the temperature of the substrate solution (800 µL) achieved 40 °C, 200 µL of enzyme solution was added, and the reaction was carried out for 6 min at this temperature. The hydrolysis of ONPG was terminated by adding 300 µL of 1 M Na_2_CO_3_ to the reaction mixture, and the absorbance was measured at 410 nm. One unit (U) of the enzyme activity denoted 1 µmol of *o*-nitrophenol liberated from the substrate in 1 min under the standard reaction conditions.

Protein concentration was determined according to Bradford’s method using the Quick Start Bradford Protein Assay kit (BioRad, Hercules, CA, USA).

SDS-PAGE of proteins was carried out on slabs (100 × 83 mm) of 12% polyacrylamide gel [87]. The samples were denatured for 5 min at 96 °C in the presence of 10% SDS and 0.5% β-mercaptoethanol. The gels were stained with Coomassie brilliant blue R-250 (BioRad, Hercules, CA, USA), according to the manufacturer’s instruction.

## 5. Patents

The host/vector expression system based on *D. macquariensis* strain D50 has been granted a patent number EP 3,530,739 A1.

## Figures and Tables

**Table 1:** The results of L_9_ orthogonal array experiments for different culture conditions on *D. macquariensis* strain D50 growth and corresponding S/N ratios.

Run	OD_660_	S/N Ratio
1	69.60	36.44
2	84.60	37.55
3	88.17	38.63
4	62.90	35.84
5	83.00	37.55
6	71.03	36.76
7	78.77	38.01
8	70.00	35.61
9	73.90	37.82

**Table 2:** S/N ratio response table for controlled factors.

Parameter	Value	Level	S/N Ratio
Temperature (°C)	20	1	37.39
	23	2	36.36
	25	3	37.06
pH	5.5	1	37.11
	6.0	2	36.91
	7.0	3	36.79
Aeration (vvm)	0.8	1	35.93
	1.0	2	36.77
	1.5	3	38.10
Stirring (rpm)	150	1	36.55
	250	2	37.04
	300	3	37.22

**Table 3:** Analysis of variance.

Source	DF	SS	MS	F-Value	*p*-Value	Contribution (%)
Model	8	2296.0	287	8.42		
Temperature	2	285.6	142.81	4.19	0.026	8.88
pH	2	205.9	102.95	3.02	0.065	6.40
Aeration	2	1367.0	683.51	20.06	0.000	42.51
Stirring	2	437.5	218.73	6.42	0.005	13.60
Error	27	920.1	34.08			28.61
Total	35	3216.1				100
**Model Summary**						
S	R^2^			R^2^ (pred)		
58.37	71.39%			49.14%		

**Table 4:** Results of *D. macquariensis* D50 transformation with *Bsp*HI-linearized pH3Bgal32d, pH4Bgal32d, pH5ABgal32d, and pH6Bgal32d plasmids.

Recombinant Yeast Strain	No. of Blue Colonies (Zeo^R^, β-Gal^+^ Phenotype)	No. of Cream-Colored Colonies (Zeo^R^, β-Gal^−^ Phenotype)
*D. macquariensis* D50/pH3Bgal32d	283	161
*D. macquariensis* D50/pH4Bgal32d	125	398
*D. macquariensis* D50/pH5ABgal32d	28	58
*D. macquariensis* D50/pH6Bgal32d	4	6

**Table 5:** The activity of *Paracoccus* sp. 32d β-d-galactosidase and protein concentration in cell-free extracts obtained in various methods of disintegration of recombinant *D. macquariensis* D50 yeast cells.

The Method of Yeast Cells Disintegration	Protein Concentration (g/L)	Activity (U/L)
Sonication	2.8	0
Enzymatic-chemical lysis using YeastBuster Protein Extraction Reagent	0.1	205
Disintegration using glass beads and lysis buffer	0.6	1150

**Table 6:** Purification of recombinant *Paracoccus* sp. 32d β-d-galactosidase.

Purification Step	Total Activity (U)	Total Protein (mg)	Specific Activity (U/mg)	Purification Fold	Yield (%)
Cell-free extract	1150	634.0	1.8	1	100
HiTrap Q FF	772	41.0	18.8	9	67
Mono Q 5/50 GL	600	16.6	36.1	18	52
Superdex 200 pg	565	12.1	46.7	23	49

**Table 7:** Comparison of selected properties of recombinant *Paracoccus* sp. 32d β-d-galactosidase produced in two different expression hosts.

Expression Host	Specific Activity (U/mg)	Optimal Temperature (°C)	Relative Activity at 10 °C (%)	Thermostability	References
*E. coli* LMG194	41.0	40	15	Up to 30 °C for 120 min	[37]
*D. macquariensis* D50	46.7	40	15	Up to 30 °C for 120 min	This study

**Table 8:** The controlled factors and their assigned levels.

No.	Factor		Level	
		1	2	3
A	Temperature (°C)	20	23	25
B	pH	5.5	6.0	7.0
C	Aeration (vvm)	0.8	1.0	1.5
D	Stirring (rpm)	150	250	300

**Table 9:** The orthogonal array (L_9_) for uncoded factors.

Run	Temperature (°C)	pH	Aeration (vvm)	Stirring (rpm)
1	20	5.5	0.8	150
2	20	6.0	1.0	250
3	20	7.0	1.5	300
4	23	7.0	1.0	150
5	23	5.5	1.5	250
6	23	6.0	0.8	300
7	25	6.0	1.5	150
8	25	7.0	0.8	250
9	25	5.5	1.0	300

**Table 10:** Oligonucleotide primers used in this study.

Name	Sequence ^1^ (Restriction Site)	PCR Product (bp)
CYCD50zaAOX1for	AAGACCGGTC TTGCTAGATT CTAA**ACCATT TATTAGATGT TGAAAAGTTT ACTCG** (*Age*I)	H3.1 (312)
CYCD50zaAOX1rev	GGGGGATCCG CACAAACGAA GG**GTTGCCAC TAATTGTAAT ATAATTGCCG TC** (*Bam*HI)	H3.1
pGsTD50_forward	AAAAGATCT**T ACCACAATTC AAGACGGCC** (*Bgl*II)	H3.2 (518)
pGsTD50_reverse	GGGTTCGAA**G TTTAATTATT AAATATTCTT ACTAGTTAAT TATATTCA** (*Bst*BI)	H3.2
pTEF1pH4_forward	TGCGGATCC**A CTTCTCTTCT TACTTTCACT CCTTTCCCTA C** (*Bam*HI)	H4 (370)
pTEF1pH4_reverse	ATGATTAATT GTCAACACCG CC**TTTTAATT AAGTTTAGTT TAGATGAAGT AAAAGAAG** (*Ase*I)	H4
BleoRCpGfree_For	CTAAACC**ATG G****CCAAGTTGA CCAGTGCTGT CCCAGTG** (*Nco*I)	H4.1 (390)
BleoRCpGfree_Rev	TTTACGTG**TC AGTCCTGCTC CTCTGCCACA AAGTGCAC** (*Bse*RI)	H4.1
CYC1G12_For	GTCGATGATA TC**ATTTAGAA CACTCTAGAT AAGCAAAAG** (*Eco*RV)	H5.1 (588)
CYC1G12_Rev	TTGCTCACAT GT**ATTATAAT GTAAGCGAAG TTTGAC** (*Pci*I)	H5.1
pIEF2pH6_forward	TGCGGATCC**C ACTACTCAGA CTTACCACCG CATATACAG** (*Bam*HI)	H6 (684)
pIEF2pH6_reverse	ATGATTAATT GTCAAC**TTTG TTTAATGTAT AATAATAGTA TACTGTATTG** (*Ase*I)	H6
F32dBgalXho	ACTGCTCGAG AAAAGA**ATGC GGGTGACCCA GAAACTGAAC CATG** (*Xho*I)	H346_Bgal32d (2222), H5A_Bgal32d (2223)
R32dBgalXba	AGTCTCTAGA **CTAGCCGACG GTGACCGTGG CCAC** (*Xba*I)	H346_Bgal32d
R32dBgalNot	TTATGCGGCC G**C****TAGCCGAC GGTGACCGTG GCCAC** (*Not*I)	H5A_Bgal32d
2F32dNested	**CCGAAGGAGAGGGCGAGCTGA**	Bgal32dNested (963)
R32dNested	**TCGGCTCTCGCCAGATGTCAA**	Bgal32dNested

^1^ Sequences complementary to the template are boldfaced. Restriction sites used for cloning are underlined.

## Data Availability

Data can be obtained from the corresponding author upon reasonable request.

## Supplementary Materials

The following supporting information can be downloaded at: https://www.mdpi.com/article/10.3390/ijms231911691/s1.
