# Supplementary material for: A New Expression System Based on Psychrotolerant Debaryomyces macquariensis Yeast and Its Application to the Production of Cold-Active β-d-Galactosidase from Paracoccus sp. 32d
Source: Int J Mol Sci. 2022 Oct 2;23(19):11691. doi: 10.3390/ijms231911691 (PMC9569826; doi:10.3390/ijms231911691)
Supplement: Supplementary file 1 [file ijms-23-11691-s001.zip › ijms-1916413-supplementary.pdf]

1 tggtgcatac gtcagtctgg gtgtgtagtc acagcaatcg cagccaaaat gggctcatct
61 gccacatggt tctcttcgct gcgtcagacg gcccttaatt tcatccagaa acttccatat
121 tgcccctgca atacaattga gtccttggat tgtttttctc gcctccgtac ggcgtactgc
181 cgtcgagtac gctctgcgac acattccgat ctacaatttt tcacagccat aattcaggga
241 cctttttgta tactggtcta tttcaaatga gacaactcgc tttccaaccc catataaccg
301 tctatctact tcatgggtca tcatgcattt gtttccaact ttctacacct atttgttaaa
361 caatagtact aatattgcta taggtgtaga tcagagaact ctccagcgta aatttcagaa
421 cctatgagaa atttttttc aggaaaaatt ttctatgaga ctcatctctg ggctccttgt
481 tggatcttta tataaggaac actagaatcc cttaattttt aattgaatta caaaaatttt
541 tttctataca tatatctcat atattaatta atcaaa**atgg gtaaagaaaa gactcacgtt**
601 **aatctcgttg tcattggtca cgtcgattct ggtaaatcta ctaccaccgg tcacttaatt**
661 **tacaagtgtg gtggtatcga taagagaacc attgaaaagt tcgaaaagga agccgctgaa**
721 **ttaggtaagg gttctttcaa gtacgcctgg gttttagaca agttaaaggc tgaaagagaa**
781 **agaggtatca ccattgatat cgctttatgg aagttcgaaa ctccaaagtt ccacgtcacc**
841 **attattgatg ctccaggtca cagagatttc atcaagaaca tgattactgg tacttcccaa**
901 **gctgattgtg ctattttgat tattgctggt ggtattggtg aattcgaagc cggtatctcc**
961 **aaggatggtc aaaccagaga acacgcttta ttagcttaca ccttaggtgt taagcaattg**
1021 **attgttgcta tcaacaagat ggactctgtc aaatgggaca aggccagatt cgacgaaata**
1081 **gtcaaggaaa cctctaactt cgtcaagaag gtcggtttca acccaaagag tgttccattc**
1141 **gtcccaatct ctggttggaa cggtgacaac atgattgagc catcctctaa ctgtccatgg**
1201 **tacaagggtt gggaaaagga aaccaaggct ggtaagtcct ctggtaagac tttgttagaa**
1261 **gctattgatg ctatcgaacc accaaccaga ccaactgaca aggctttaag attaccattg**
1321 **caagatgtct acaagatcgg tggtattggt actgtgccag tcggtagagt tgaaaccggt**
1381 **atcatcaagg ccggtatggt tgtcaccttc gccccagctg gtgttaccac tgaagtcaag**
1441 **tccgttgaaa tgcatcacga acaattaacc gaaggtgttc caggtgacaa cgttggtttc**
1501 **aacgtcaaga atgtttccgt taaggaaatc agaagaggta acgtctgtgg tgactccaag**
1561 **aacgacccac caaagggtgc tgactctttc accgctcaag ttattgtctt aaaccatcca**
1621 **ggtcaaatct cctctggtta ctctccagtc ttagattgtc acactgctca cattgcttgt**
1681 **aagttcgaca ctttaatcga aaagattgac agaagaactg gtaagaagtt agaagacaac**
1741 **cctaagttca tcaagtctgg tgatgctgct attgtcaaga tggtcccatc taagccaatg**
1801 **tgtgttgaag ctttcactga ctacccacca ttaggtagat tcgctgtgag agatatgaga**
1861 **caaactgttg ctgtcggtgt tatcaagtct gttgaaaagt ctgacaagtc cggtaaggtt**
1921 **actaaggctg ctcaaaaggc tgctaagaaa taa**gctcctt aatttgaaat tgtccgggag
1981 aagctacttg aatttgaatt acgtaaattt cgagtttttt ccctacagtt agaattctgt
2041 ttattttgat aagtaatttt atactttaat gtttaatagt tgatctaaac gctcctttgt
2101 aggaacctat ccaatttgaa cgcaaactct attcggactg cagtatacac agttcccgtt
2161 gacctcaatc gtcggatatt atgtccaaca tcagtcgatg ccatgaacac aaaagccgtc
2221 cgaaaaattt ccttatcaag ccacatgtga aaagtcgtcc tcaatgacga gtcaaagatc
2281 tgataagccc accaaactta tattaagatc cctgttatat gaagttccat ttttcacata
2341 ttctttaaca cagctggtac cctcaactgt ttcaaacttt gagatggatt agtattgcgt
2401 tattgtaggt agaaaagtta gtgaaaaatc atcattgaac cgagtcataa tctgggaaa
2461 cagagtcagt aaaataccgt gttaaatatt tttcattgac tctgagtggt acaactttcg
2521 tattagccta tgaagtattt aaaccatcga gaatcctttt aaaatgacaa taaagtagtt
2581 ttaatatcat tcatcttttg ataaatacaa tataatcata gaattaattt tttttgtttt
2641 ataaaccaaa aaaaaataga gaaggatgat ttcaagagtt gttgcaagat cggctcgtgc
2701 taatagattg tttaccttga ccagatctgt tccaaaggta tacaatttga acttgaaaat
2761 tgcatcgagt agcagatttt tagcttcaaa ggccgacaca tcatcagcag attatgaaaa
2821 agtttacgat ccaagtgcca agactgtgtc tttgggcaat ttggatacgt tcgccagacg
2881 tcatcttggt cctacccctg acaatgtcaa gacgatgttg agtaccttgg gatacaagga
2941 tttggacgag tttttgagca aagccatccc agagcacgtt ttatacaaaa gggcattaca
3001 gattcaacca caacaaggtt acactgagct ggaaatgttg gaacatttgc aaaaattggc

Figure S1: Nucleotide sequence of the *D. macquariensis* strain D50 *TEF1* gene (bold text) with 5′ and 3′-flanking regions. The putative promoter sequence is red.

1 cactactcag acttaccacc gcatatacag acattaacca taatagtgcc atctacaggg
61 cacaatgcat ttaatacaca atctaatata cagcttaagt agagcccatc tgtaattaaa
121 cccacacaaa gagtaattca gtcacatata ctaatgtgaa cggcattcat ttgacctagt
181 gtcaacatgg caaaagcttc cgccgcagtt tcgcaatttc actacatgtc gcctgtcatc
241 agctacgaac tgctatgtaa gatcaactgc aaaatgttcc actggtccat aatcgaagtt
301 tctaggagtt tgccaaaaaa tttagtccca tactatccca accaaatgtc ctcaaataac
361 taccatcaca cctattttgc caagttcttc ctatctacta tatagctact tcatgctgct
421 tgcaataccg tagtctacgg ccatagcact tggattctga atacttagag gaaatagggt
481 aaagccctat ccgggtaaat taaatatttg tcctgctgaa aaaaaaaatt tcgccacgac
541 accctctgat gaatggaacg caattggctc tgcaaaaatt tataaggaat taaattgaag
601 aacttctttt tccttctctt tttttcaata cagtatacta ttattataca ttaaacaaa**a**
661 **tgggtaaaga aaagactcac gttaatcttg ttgtcattgg tcacgtcgat tccggtaaat**
721 **ctaccactac cggtcactta atttacaagt gtggtggtat cgataagaga actattgaaa**
781 **agttcgaaaa ggaagccgct gaattaggta agggttcttt caagtacgct tgggttttag**
841 **acaagttaaa ggctgaaaga gaaagaggta tcaccattga tatcgcttta tggaagttcg**
901 **aaactccaaa gttccacgtc accattattg atgctccagg tcacagagat ttcatcaaga**
961 **acatgattac tggtacttcc caagctgatt gtgctatttt gattattgct ggtggtattg**
1021 **gtgaattcga agctggtatc tccaaggatg gtcaaaccag agaacacgct ttattagctt**
1081 **acaccttagg tgttaagcaa ttgattgttg ctatcaacaa gatggactct gtcaaatggg**
1141 **acaaggccag attcgacgaa atcgtcaagg aaacctctaa cttcgtcaag aaggtcggtt**
1201 **tcaacccaaa gagtgttcca ttcgtcccaa tctctggttg gaacggtgac aacatgattg**
1261 **aaccatcatc taactgtcca tggtacaagg gttgggaaaa ggaaaccaag gctggtaagt**
1321 **cctctggtaa gactttgtta gaagctattg atgctatcga accaccaacc agaccaactg**
1381 **acaaggcttt aagattacca ttgcaagatg tctacaagat cggtggtatt ggtactgtgc**
1441 **cagtcggtag agttgaaacc ggtattatca aggccggtat ggttgtcacc ttcgccccag**
1501 **ctggtgtcac cactgaagtc aagtccgttg aaatgcatca cgaacaatta accgaaggtg**
1561 **ttccaggtga caacgttggt ttcaacgtca agaacgtttc cgttaaggaa atcagaagag**
1621 **gtaacgtctg tggtgactcc aagaatgacc caccaaaggg tgctgactct ttcaccgctc**
1681 **aagttattgt cttaaaccat ccaggtcaaa tctcctctgg ttactctcca gtcttagatt**
1741 **gtcacacagc tcacattgct tgtaagttcg acactttaat cgaaaagatt gacagaagaa**
1801 **ctggtaagaa gttagaagac aaccctaaat tcatcaagtc tggtgatgct gctattgtca**
1861 **agatggttcc atctaagcca atgtgtgttg aagctttcac tgactaccca ccattaggta**
1921 **gattcgctgt cagagatatg agacaaactg ttgccgtcgg tgttatcaag tctgttgaga**
1981 **agtctgacaa gtccggtaag gttaccaagg ctgctcaaaa ggctgctaag aaataa**gctg
2041 attaatgtag aattttatat tggttacttg ttttatatta ggctttttga atacatatga
2101 actttaaaaa tgattgtagc ctccgataag cactagctta ttgtctctgt aaatcgttat
2161 gtggcgtggc caagtatctg gcgcatgcac atttatcgct aaagactatt taatagaatt
2221 tgaacttcac actggcgaac cctactagtt aaacgacttt tatacttttc tccgagtaat
2281 aacaaagaac gtacctgacg ttataatcaa catatgccat atcaccttct aggtccttag
2341 gtattaatta atagcatggt gaaaattttt ttagcacaat agatactgta tacaaccaga
2401 tatcatgttc aactggaaat agatacagtt aaatgaactt tgttgataga gggtctattt
2461 gacatagaac cgtgttgacg ataagc

Figure S2: Nucleotide sequence of the *D. macquariensis* strain D50 *IEF2* gene (bold text) with 5′ and 3′-flanking regions. The putative promoter sequence is pink.

```
   1 tcgtacgtta cattatatac actcctatat atgaagtaga ccaattcttt aacactgtga
  61 ccaacatcgt ttctgtacgt ctattggtcg aattcgccga aatatacata cttcctgtat
 121 atggtcagaa tcttcacatc ggtgttatta ggcgacaccg aaacccttaa acccccgctt
 181 cgcactcggt cggtcgtgtc tgggtaacaa atgcgttacg ctacacccat tcgctaaatc
 241 caaacgtcat tccataggtc gggtccgacg aaagcctcac ttccgcacac cacatccatt
 301 catcaggatc tgacttcgcg cagacgtcat cgacccctgg ggctcgtgtt cggtcttcgc
 361 ttcaatccca gggtgcgcgg tctttttccc ctctcccgga cgacctatca taccacaatt
 421 caagacggcc cttatgtgcc tgttctccaa cgtgtcctta tcagttcata atcgccacct
 481 ggttgtgtct caatcgccag atttaccggt tctcctaaat tgcgcctaca tttgtcgttc
 541 tcacgtcctg tgctctcttt ctacatggac gagttgctgc caacgaccat ttttcacggt
 601 atcaagtggt ggttagcctc ccggcgaccc tggacggctc gcttccgttg atccttccat
 661 actaattaac ggcctcggac ttttgtagcg cgaaatcatg agaatcacca aactcccggc
 721 aactagctcg gtcgtgcccg gcaccggggc ctctatggga ttcttcgccc cgggagtcca
 781 gcaataagaa catagtggaa aattataaat atgagtaatt cgccgtgcaa tcgacatgga
 841 gtatatttta aaggttatct tattattcaa ttgaatataa ttaactagta agaatattta
 901 ataattaaac atggctatca ctattggtat taacggtttc ggtcgtattg gacgtttagt
 961 cttaagagtc gctttagaaa gatctgacat caaggttgtt gccgtcaatg atccattcat
1021 tgccgctgaa tacgctgctt acatgttcaa gtacgattct actcacggta gatacaaggg
1081 tgatgtaagc tccaaggacg acaccttagt catcgacggt caatctatca aggttttcgg
1141 tgaaagagac ccagcctcga ttccatgggg taaggtcggt gttgactacg tcatcgaatc
1201 caccggtgtc ttcaccacta tggaaggtgc tcaaaagcac attgacggtg gtgccaagaa
1261 ggtcattatc actgctccat cctcagatgc tccaatgttc gttgttggtg ttaacgaaca
1321 aaaatacacc ccagacttga agattgtttc caacgcttct tgtactacta actgtttggc
1381 tccattagcc aaggttatca acgacacctt cggtattgaa gaaggtttaa tgaccactgt
1441 ccactccatc actgccaccc aaaagaccgt tgatggtcca tcccacaagg actggagagg
1501 tggtagaacc gcttctggta acattatccc atcttccacc ggtgctgcca aggcggtcgg
1561 taaggttatt ccagaattaa acggtaagtt gaccggtatg tctttgagag ttcctactgt
1621 cgatgtttca gttgttgatt taaccgtcag attgaagaaa cctgctacct acgaagaaat
1681 ctccgaagct atcaagtctg cttctaacgg tgaattaaag ggtgtcttag gttacactga
1741 agatgctgtt gtttccaccg atttcttagg ttccagctac tcttctatct tcgatcaaaa
1801 ggccggtatc ttattatccc caaccttcgt caagttgatc tcttggtacg ataacgaatt
1861 cggttactcc accagagttg tcgatttatt agaacacgtc gccaaggctt ctaattaact
1921 agccgaccct aaactataat ttatttatgg tttatcgtcc ctagcatata tatgcgcaat
1981 aaaatgtatt tattttaaca ttaaaacact tgtatatgta gcgagtttgg catgtatgtg
2041 aattcttagg ctcgtatggg tttccaaccg tagctggtgt aggtgtgatg tacttttaaa
2101 ctatattaac cgttctaata tcttgtgcgt atttatttgt aaatattcac atatttgttt
2161 aaattctata catatacatt atcactatca catacattat cactattata tacacagtgc
2221 atatttcctt attacctaaa tttgacactg ctcctcgttg ttacaacggt ttctcttcat
2281 ggacaaatta tttctagatc aaacattcaa attatacaaa tcaaatgata ctgtacctca
2341 cttgactcaa caacaattgg actatatctc aacaataaca tgtcaatatt tattccaaac
2401 gttggttaat gatatgatat cagattacag atcatttgag gatttactcc tagattttaa
2461 aatcactcat agctcgagac agtttaatca atttacaatc acatatattt ccataagaca
2521 caagggatta ttgcctcgac aaaccataat attcatcaaa gctactgata ctaacgacaa
2581 cgcatcatca ttcaatacgt tggtgatgat aaaaacaacg aactcatcct tattaaggat
2641 tctaattcag gcgattgaaa atctacctac agaagttccc ataatagtac gggaattaaa
2701 attcaacgag aaatatctca acttaattat aaataatgta tctaacagct tattatctat
2761 agataattat gctgaggtca taggtgacgt tgaattgatt tatcaaacta gccacctcgt
2821 atcgaacgat tcattaagaa acatcatcat caatattccc gaaaaagatg taagtattat
2881 atcggattcc aaaggactca ttaataacgt caatgatttc ttgaaacaaa cgagcctgtt
2941 aaattttaat aacctacctt tgattcggtt cacttcaagg
```

Figure S3: Nucleotide sequence of the *D. macquariensis* strain D50 *GAP* gene (bold text) with 5′ and 3′-flanking regions. The putative promoter sequence is green.

1 aggaattaca aatagaagcc ataaatatat tcattcctgt atataaatat tagccaataa

61 aattgcattc gttaacaatt atgagaaatt tttcaccttg attggaacat atataaaagg

121 aagctttctg gcccgattca aaacaagttg ttaattccca taatataatc ttttatattt

181 taaccaggtt tcttaacata tatc**atgcca gctccatttg aaaaaggttc cgaaaagaag**

241 **ggtgctaact tatttaaaac cagatgttta caatgtcaca ctgttgaaga aggtggtcca**

301 **cacaaggttg gtccaaactt acacggtatt attggtagaa catctggtca agccgctggt**

361 **tactcttaca ctgatgccaa taaaaagaag ggtgttcaat ggactgaaca aactttatcc**

421 **gactacttgg aaaacccaaa gaaatatatc ccaggtacca agatggcctt cggtggttta**

481 **aagaaaccta aggacagaaa cgatttgatc acctatatag ccgaagctac caaataa**acc

541 atttattaga tgttgaaaag tttactcgtc gttttgtaca aacactgttt aatgggcctc

601 gaatttagct agttacacat ttatctgtat atacatgaat aatattaacg aacgatgtta

661 gattaaatgc ttacagtaga gtacaagcag tggggttat atttggatgg acattatctg

721 ttcacgtgac ccagtatgag tgaaaagttc gccaagtcaa ccactgtatt acagacggca

781 attatattac aattagtggc aac

Figure S4: Nucleotide sequence of the *D. macquariensis* strain D50 *CYC1* gene (bold text) with 5′ and 3′-flanking regions. The putative transcriptional terminator sequence is purple.

```
   1 atatttatta aaagaaaaga aatagatcaa gattaatcca aggttgttta aaagaagaca
  61 agacatgtaa cgagttgaat tttataataa aaaaagaaaa tatatgattt aaagtataca
 121 tgaaattgta gaaggtatgc gtttagaaag gagctgaagt taacgagatg cattcaattt
 181 caaagacaga gagttcttac atttatctta gacgaattag gatgcttcat tgtagtagat
 241 ttctctgatt tggtgcatgg gttgctttat tgttgcaaaa tagtgggga gaggggaaga
 301 ggaaagataa acattatatg caggtcactt aaacacttga taaagtcatc ataaaacaac
 361 ctaaatcaac tatttgagtc caaaatttta cataaattac ataaattact ttttgaaatt
 421 ctttcaatat acagttcaaa taataaaaat tagggttagc gtagataaaa cgaatattgt
 481 aggtttatgt tgggcggtaa attacacaaa gttgagaaga taagatgcgc tacaaagtga
 541 aaaaaataaa aaattattga tctgaagatt tttttttttg gagatttcgg aaaattttga
 601 ggtctcatcg acttatcgat ccgccaaagt gtaacctaaa tagtgaaacc taaacccaaa
 661 taactactag gttatagtaa agacgattga gtaagtcagg attgggataa aaaaataatt
 721 gaatcaaata ctctacatga atttattttc ccctctaagt ctcatttctc attttatatt
 781 tacatttata tttcataacc tctctttaaa cttctcttct tactttcact cctttcccta
 841 cacggttatt gtacgtgtct attccggtat ttaaattagc tatgctgaaa attacgacag
 901 aatgttttta ttgccgcgtg tattttcaat tatatagtag tatttaagca tcgtcgacct
 961 gcacaaaacc ctcatcgtac catattttac ccatactagt tgaaaatttt tttttttttt
1021 gctctgcggg tgagggggcg ttgcccaata tataaggagt ggcccttgtc ttagacaaaa
1081 attttccagg ttataagttt tttttttttc ttcttctttt acttcatcta aactaaactt
1141 aattaaaaat gggtaaagat aagaatcacg ttaacgtcgt cgtcattggt cacgtcgatt
1201 ccggtaagtc aaccactact ggtcacttaa ttttcaagtg tggtggtatt gataagagaa
1261 ctattgaaaa gttcgaaaag gaagctaatg aattaggaaa aggttctttc aagtacgctt
1321 gggttttaga caagcttaag gctgaaagag aaagaggtat cactattgat attgctttat
1381 ggaagttcga aactccaaag tatcacgtta ctgttattga tgctccaggt cacagagatt
1441 tcattaagaa tatgattact ggtacttctc aagctgattg tgctgtttta attattgctg
1501 gtggtattgg tgaattcgaa gccggtattt ctaaggatgg tcaaaccaga gaacatgctt
1561 tattagctta caccttaggt gttaaacaat taattgttgc tgtcaacaag atggattccg
1621 ttaaatggga taaggctaga tttgaagaaa tttcaaagga aacctctaat ttcgttaaga
1681 aggttggtta caatccaaag actgttccat tcgttccaat ctctggttgg aatggtgata
1741 acatgattga accatctgct aactgtccat ggtacaaggg ttgggaaaag gaaaccaagg
1801 ccggtaaggt cactggtaag actttattag atgctattga tgctattgaa ccacctcaaa
1861 gaccaactga taagccatta agattaccat tacaagatgt ttacaagatt ggtggtattg
1921 gaactgtgcc agttggtaga gttgaaaccg gtatcattaa ggctggtatg gttgttgttt
1981 tcgccccagc tggtgttacc actgaagtca agtccgttga aatgcatcat gaacaattag
2041 ttgaaggtgt tccaggtgac aatgttggtt tcaatgttaa gaatgtttcc gttaaggaaa
2101 ttagaagagg taacgtttgt ggtgacacca aggttgatcc accaaaggct gctgcttcat
2161 tcaatgctca agttattgtc ttaaaccatc caggtcaaat ttctgctggt tactctccag
2221 ttttagattg tcacaccgcc catattgctt gtaagttcga tcacttagtt gaaaagattg
2281 atagaagaac tggtaagtca atggaagatg aaccaaaatt catcaagtcc ggtgatgctg
2341 ctattgttag aatggtccca tccaagccaa tgtgtgttga agctttcact gactacccac
2401 cattaggtag attcgctgtt agagatatga gacaaactgt cgctgtcggt gttatcaagt
2461 ctgttgacaa gtccgacaag gttggtaagg tcaccaagtc tgctgttaag gctgctaaga
2521 agtaaacaac ctagtttacc tttgatgtaa gtagtttttc tttgactaat gtatgaaatt
2581 aaatatatat aaaaaaaaag tagaaatgag cgtattatgt aactgtttgc tgcgaaaaat
2641 cgtattatag gtgaattact gtgaaatatg tcttaatatt tatgtcactc gagccacaaa
2701 tgtgatttat gattttatta aacaaaagag tataatgtga tatttaggat aaaacaaaat
2761 ttggcatgtt aagggttcta agaggtttat atctatgcac ggagaaaaaa gtaaaagaag
2821 ttcatagtgt tgcagcgaat aaatttttg aataaaatca atcggaatta gtaaccttct
2881 tttatgcagc taaattatat taaagggtac ctacagctga gttggattac gttggaatgt
```

Figure S5: Nucleotide sequence of the *C. santamariae* strain G12 *TEF1* gene (bold text) with 5′ and 3′-flanking regions. The putative promoter sequence is blue.

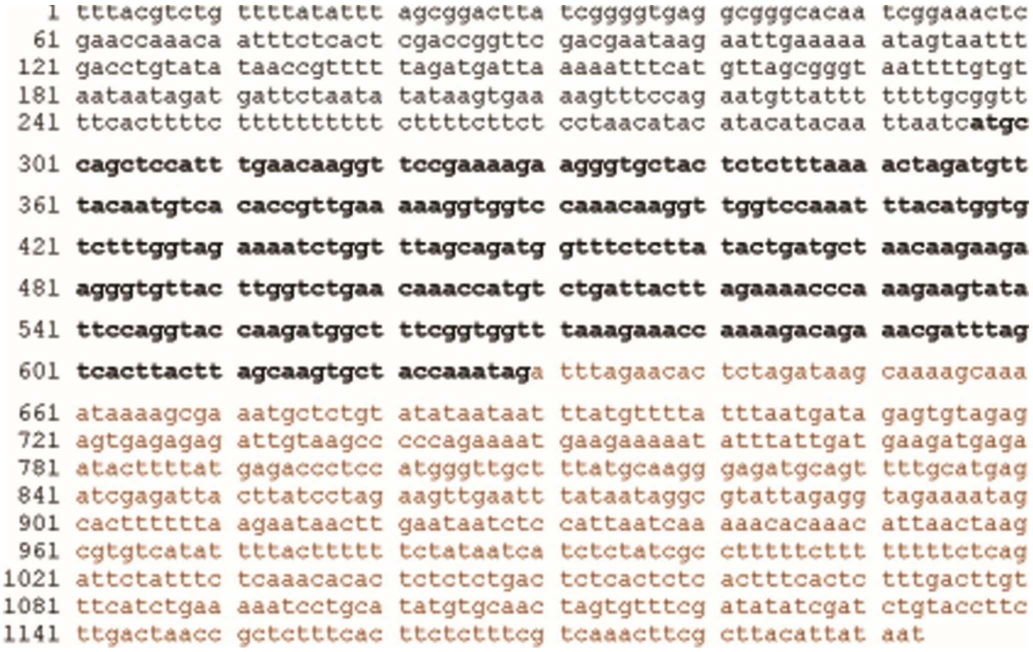

Figure S6: Nucleotide sequence of the *C. santamariae* strain G12 *CYC1* gene (bold text) with 5′ and 3′-flanking regions. The putative transcriptional terminator sequence is brown.

Table S1. Extracellular proteolytic activity and concentration of extracellular proteins of yeast strains isolated from soil samples collected from various sampling sites in Antarctica [1] after 72 h of cultivation.

Yeast strain	Proteolytic activity at pH 7.0 (nmol/mL × min)	Proteolytic activity at pH 4.0 (nmol/mL × min)	Extracellular proteins concentration (mg/mL)
Debaryomyces macquariensis D50	6.30	7.50	0.030
Debaryomyces hansenii IBT-D1	6.97	3.20	0.032
Goffeauzyma gastrica IBT-D7	21.11	8.68	0.060
Goffeauzyma gilvescens IBT-D13	16.18	8.56	0.096
Goffeauzyma gilvescens IBT-D15	11.45	12.36	0.112
Goffeauzyma gilvescens IBT-D16	11.84	14.54	0.136
Goffeauzyma gilvescens IBT-D20	15.98	24.47	0.122
Goffeauzyma gilvescens IBT-D60	13.02	15.79	0.178
Leuconeurospora sp. IBT-D59	38.63	17.70	0.362
Naganishia globosa IBT-D37	17.76	8.90	0.158
Naganishia adeliensis IBT-D58	11.45	12.43	0.215
Naganishia albida IBT-D62	27.60	18.30	0.329

Rhodotorula mucilaginosa IBT-D12	28.22	17.50	0.150
Rhodotorula mucilaginosa IBT-D34	18.55	12.17	0.105
Rhodotorula mucilaginosa IBT-D38	18.29	8.29	0.099

[1] Białkowska, A.M.; Szulczewska, K.M.; Krysiak, J.; Florczak, T.; Gromek, E.; Kassassir, H.; Kur, J.; Turkiewicz, M. Genetic and biochemical characterization of yeasts isolated from Antarctic soil samples. *Polar Biol.* **2017**, *40*, 1787-1803, doi: 10.1007/s00300-017-2102-7.
